# A Novel Vaccine Strategy Employing Serologically Different Chimpanzee Adenoviral Vectors for the Prevention of HIV-1 and HCV Coinfection

**DOI:** 10.3389/fimmu.2018.03175

**Published:** 2019-01-18

**Authors:** Felicity Hartnell, Anthony Brown, Stefania Capone, Jakub Kopycinski, Carly Bliss, Shokouh Makvandi-Nejad, Leo Swadling, Emma Ghaffari, Paola Cicconi, Mariarosaria Del Sorbo, Roberta Sbrocchi, Ilaria Esposito, Ventzislav Vassilev, Paula Marriott, Clair M. Gardiner, Ciaran Bannan, Colm Bergin, Matthias Hoffmann, Bethany Turner, Alfredo Nicosia, Antonella Folgori, Tomáš Hanke, Eleanor Barnes, Lucy Dorrell

**Affiliations:** ^1^Nuffield Department of Medicine, University of Oxford, Oxford, United Kingdom; ^2^ReiThera s.r.l., Rome, Italy; ^3^GlaxoSmithKline Vaccines, Brussels, Belgium; ^4^Centre for Clinical Vaccinology and Tropical Medicine, Jenner Institute, University of Oxford, Oxford, United Kingdom; ^5^School of Biochemistry and Immunology, Trinity College, Dublin, Ireland; ^6^St James' Hospital, Dublin, Ireland; ^7^Division of Infectious Diseases and Hospital Epidemiology, Kantonsspital St Gallen, St Gallen, Switzerland; ^8^Keires AG, Basel, Switzerland; ^9^Department of Molecular Medicine and Medical Biotechnology, University of Naples Federico II, Naples, Italy; ^10^CEINGE-Biotecnologie Avanzate, Naples, Italy; ^11^Jenner Institute Laboratories, University of Oxford, Oxford, United Kingdom; ^12^International Research Center for Medical Sciences, Kumamoto University, Kumamoto, Japan; ^13^Oxford NIHR Biomedical Research Centre, Headington, United Kingdom

**Keywords:** HIV-1, HCV (hepatitis C virus), vaccine, coadministration, clinical trial, conserved region, non-structural protein (NS), transcriptomics analysis

## Abstract

**Background:** Nearly 3 million people worldwide are coinfected with HIV and HCV. Affordable strategies for prevention are needed. We developed a novel vaccination regimen involving replication-defective and serologically distinct chimpanzee adenovirus (ChAd3, ChAd63) vector priming followed by modified vaccinia Ankara (MVA) boosts, for simultaneous delivery of HCV non-structural (NSmut) and HIV-1 conserved (HIVconsv) region immunogens.

**Methods:** We conducted a phase I trial in which 33 healthy volunteers were sequentially enrolled and vaccinated via the intramuscular route as follows: 9 received ChAd3-NSmut [2.5 × 10^10^ vp] and MVA-NSmut [2 × 10^8^ pfu] at weeks 0 and 8, respectively; 8 received ChAdV63.HIVconsv [5 × 10^10^ vp] and MVA.HIVconsv [2 × 10^8^ pfu] at the same interval; 16 were co-primed with ChAd3-NSmut [2.5 × 10^10^ vp] and ChAdV63.HIVconsv [5 × 10^10^ vp] followed at week 8 by MVA-NSmut and MVA.HIVconsv [both 1 × 10^8^ pfu]. Immunogenicity was assessed using peptide pools in *ex vivo* ELISpot and intracellular cytokine assays. Vaccine-induced whole blood transcriptome changes were assessed by microarray analysis.

**Results:** All vaccines were well tolerated and no vaccine-related serious adverse events occurred. Co-administration of the prime-boost vaccine regimens induced high magnitude and broad T cell responses that were similar to those observed following immunization with either regimen alone. Median (interquartile range, IQR) peak responses to NSmut were 3,480 (2,728–4,464) and 3,405 (2,307–7,804) spot-forming cells (SFC)/10^6^ PBMC for single and combined HCV vaccinations, respectively (*p* = 0.8). Median (IQR) peak responses to HIVconsv were 1,305 (1,095–4,967) and 1,005 (169–2,482) SFC/10^6^ PBMC for single and combined HIV-1 vaccinations, respectively (*p* = 0.5). Responses were maintained above baseline to 34 weeks post-vaccination. Intracellular cytokine analysis indicated that the responding populations comprised polyfunctional CD4^+^ and CD8^+^ T cells. Canonical pathway analysis showed that in the single and combined vaccination groups, pathways associated with antiviral and innate immune responses were enriched for upregulated interferon-stimulated genes 24 h after priming and boosting vaccinations.

**Conclusions:** Serologically distinct adenoviral vectors encoding HCV and HIV-1 immunogens can be safely co-administered without reducing the immunogenicity of either vaccine. This provides a novel strategy for targeting these viruses simultaneously and for other pathogens that affect the same populations.

**Clinical trial registration:** https://clinicaltrials.gov, identifier: NCT02362217

## Introduction

Hepatitis C virus (HCV) and human immunodeficiency virus type 1 (HIV) are each responsible for significant global burden of disease and premature death. Between 50 and 80% people with acute HCV develop chronic infection and an estimated 71 million people are currently living with viraemic infection, which is transmissible and carries the risk of long-term complications, including fibrosis, cirrhosis and hepatocellular carcinoma (1, 2). Combinations of direct-acting antiviral agents now achieve cure in >90% patients but their high cost is prohibitive in both high and low resourced settings, with global treatment coverage currently at only 13% (3, 4). Re-infection after successful treatment is common in some populations (5).

Nearly 37 million people are living with HIV (PLWH), of whom more than half are now accessing effective antiretroviral therapy (ART). Scale up of access to ART and earlier initiation of treatment have led to spectacular gains in life expectancy and reductions in new infections, even in high prevalence regions (6). However, therapy is life-long and does not provide a cure, placing a considerable burden on patients and healthcare systems. Co-infection with HCV and HIV affects 2.6 million people and the incidence of HCV infection in HIV-positive men who have sex with men is increasing (7–9). Progression to end-stage liver disease (ESLD) is faster than in HCV mono-infection and ESLD is now the leading cause of death in PLWH (10, 11).

Effective preventive vaccines would greatly strengthen the current primary prevention strategies of “test-and-treat” and pre- and post-exposure antiviral prophylaxis and would be cost-effective for both HCV and HIV infections (12–14). Given the overlapping epidemiology of HIV and HCV, strategies to prevent co-infection are needed. However, significant obstacles have hampered progress: a common feature of these viruses is antigenic variability, which is driven by high rates of viral replication and error-prone reverse transcription and which enables frequent immune escape. The tissue tropism of HCV and the persistence of latent reservoirs of replication-competent HIV genomes each pose unique challenges for immune clearance. Furthermore, the immune correlates of resolution of HCV and long-term control of HIV are not fully defined.

Co-administration of vaccines against multiple pathogens is fundamental to the success of childhood and adolescent immunization programmes. As repeated doses are required to achieve adequate immunity, many licensed vaccines are administered as mixtures to ensure maximal population coverage. The potential disadvantages are increased reactogenicity and immune interference, with the latter posing a risk of inadequate protection against one or more of the targeted pathogens (15). However, several recent clinical trials have shown no immune interference when vaccines for new indications are co-administered with Expanded Programme on Immunization vaccines, for example, tuberculosis (M72/AS01) and malaria (chimpanzee adenovirus 63 and modified vaccinia Ankara encoding multiple epitope string thrombospondin-related adhesion protein) vaccine candidates, nor with co-administration of rotavirus and measles-rubella vaccines in infants and herpes zoster and influenza vaccines in adults (16–19).

The development of vaccines for HCV and HIV has focused on the induction of antibodies, preferably with broadly neutralizing activity (bnAbs) and T cell responses (20, 21). bnAbs are required for sterilizing immunity but have so far proved difficult to elicit with conventional immunogens. CD4^+^ and CD8^+^ T cells contribute to spontaneous clearance of HCV and to long-term control of HIV (22–24). Non-human primate studies have shown that live attenuated and replication-defective viral vectors elicited the most potent virus-specific T cells in the simian immunodeficiency virus (SIV) model; furthermore, vaccine-induced T cells could abort infection at an early stage in pathogenic challenge models of SIV and HCV (25–28). The main constraints on their use are safety and tolerability and complex manufacturing requirements.

We have developed candidate vaccines for HCV and HIV employing a potent heterologous viral vector platform for antigen delivery. Priming with replication-defective chimpanzee adenoviruses (ChAds) followed by boosting with modified vaccinia Ankara (MVA) has proved to be highly efficient for induction of T cells to a wide range of transgene products in diverse populations and age groups (29, 30). We have previously reported the safety and immunogenicity in human volunteers of the novel immunogens, NSmut, comprising the entire non-structural region from an HCV genotype 1b isolate, BK, and HIVconsv, comprising highly conserved regions from HIV-1 clades A-D Gag, Pol, Vif, Env sequences (Supplementary Figure 1), when delivered by ChAd and MVA vectors (31–34). In the present study, we investigated the safety and immunogenicity of these two vaccine strategies when co-administered to healthy volunteers at low risk of HCV or HIV infections.

## Methods

### Participants

Healthy male and non-pregnant female volunteers aged 18–50 were invited to participate. All study visits and vaccinations were performed at a single site in Oxford, UK.

### Ethics and Regulatory Approval

This study was carried out in accordance with the recommendations of UK National Research Ethics Service (NRES Committee South Central—Oxford A 14/SC/0195) and the UK Medicines and Healthcare Products Regulatory Agency (Eudract no. 2014-000730-30). All subjects gave written informed consent in accordance with the Declaration of Helsinki. The protocol was approved by the NRES Committee South Central—Oxford A. The study was registered with ClinicalTrials.gov (NCT02362217) and conducted in accordance with ICH-GCP. ICH-GCP compliance was independently monitored by the University of Oxford Clinical Trials and Research Governance office. A multinational independent data safety monitoring committee (DSMC) provided safety oversight.

### Study Design and Vaccinations

In this open-label study subjects were enrolled sequentially in one of three groups. Group 1 (*n* = 9) received ChAd3-NSmut (2.5 × 10^10^ vp) and MVA-NSmut (2 × 10^8^ pfu) at weeks 0 and 8, respectively; Group 2 (*n* = 8) received ChAdV63.HIVconsv (5 × 10^10^ vp) and MVA.HIVconsv l (2 × 10^8^ pfu) at the same interval, respectively). The dose of ChAd3-NSmut was based on data from a previous trial which showed that transgene-specific T cell responses reached a plateau at a higher dose of 7.5 × 10^10^ vp (31). ChAdV63.HIVconsv has been previously tested at two doses, 5 × 10^9^ and 5 × 10^10^ vp; the latter was found to be more immunogenic (33). Group 3 (*n* = 16) were co-primed with ChAd3-NSmut (2.5 × 10^10^ vp) and ChAdV63.HIVconsv (5 × 10^10^ vp) at week 0 followed at week 8 by MVA-NSmut and MVA.HIVconsv, each of which were given at half the dose (1 × 10^8^ pfu) used in Groups 1 and 2 to maintain an equivalent total dose of MVA (2 × 10^8^ pfu). Enrolment into Groups 2 and 3 commenced only after completion of priming immunizations in the preceding groups.

Vaccines were manufactured in compliance with Good Manufacturing Practice as described previously (32, 33). Vaccine vials were stored at −80°C until use and thawed ≤ 30 min prior to administration. All vaccinations were administered intramuscularly into the deltoid region of the arm. Group 3 subjects were administered the HCV and HIV vaccines in separate limbs.

### Assessment of Primary Endpoints: Safety and Reactogenicity

Volunteers were observed for up to 60 min following immunization. A safety review of the first three volunteers in each group was conducted by the DSMC 48 h following each vaccination, before proceeding to further vaccinations. Safety evaluations comprised the following: (i) solicited symptoms recorded by the participants on diary cards for 3 days following each vaccination, (ii) unsolicited adverse events, (iii) physical examination and (iv) monitoring of laboratory parameters, all of which were recorded at follow-up visits on day 1, weeks 1, 2, 4, 8, 8 + 1 (day 57), 9, 12, 14, 34. Local and systemic events were graded according to Grading Toxicity Tables given in the clinical protocol (adapted from Division of AIDS 2004).

### *Ex vivo* IFN-γ ELISpot Assay

IFN-γ ELISpot assays were performed with freshly isolated peripheral blood mononuclear cells (PBMC) as described previously, using an established laboratory SOP (31, 33). Peptide sets (15-mers overlapping by 11 amino acids) corresponding to the HCV NSmut immunogen (*n* = 494, BEI Resources) and the HIVconsv immunogen (*n* = 166, Genscript) were each tested in 6 pools (NSmut: ~80 peptides/pool; HIVconsv ~20–30 peptides/pool). The final peptide concentration was 3 μg/ml (NSmut) or 2 μg/ml (HIVconsv). PBMC were aliquoted at 2 × 10^5^ cells/well for all time points; at week 9 both 1 × 10^5^ and 2 × 10^5^ cells/well were tested in order to ensure that the anticipated peak response could be accurately quantified. The assay cut-off was set at >48 SFC/10^6^ PBMC for HCV NSmut assays, as used previously (31); for HIVconsv assays, a cut-off of >30 SFC/10^6^ PBMC, based on the mean + 3 SD of all mock-stimulated responses. Peptide-specific responses were defined as positive only if they exceeded the assay cut-off and were at least 3-fold greater than the DMSO control value. The magnitude of the response to each immunogen was obtained by summing the DMSO-subtracted responses to each peptide pool that met positivity criteria.

### Intracellular Cytokine Assay

Intracellular cytokine secretion by antigen-specific T cell was analyzed using multiparameter flow cytometry. Briefly, cryopreserved PBMCs were thawed, washed and rested overnight in R10 medium at 37°C in a humidified incubator. Cells were stimulated with HCV NSmut (1 μg/ml), or HIVconsv peptide pools (2 μg/ml), mock control (0.45% DMSO) and positive controls (SEB, 5 μg/ml; CMV pp65, 2 μg/ml, NIH AIDS Reagent Repository) at 37°C for 6 h in the presence of Golgiplug, Golgistop (BD Biosciences) and CD107a BV421. Following viability and surface staining, cells were then fixed using BD cytofix/cytoperm solution according to the manufacturer's instructions and intracellularly stained using reagents as listed in Supplementary Table 1. At least 10,000 viable singlet CD3^+^ CD4^+^ or CD8^+^ lymphocyte events were acquired using a BD Fortessa X20 cytometer. Data were analyzed using FlowJo v9.9.3 (FlowJo, US) and GraphPad Prism v7.0.

### HLA Class I-Peptide Pentamer Staining

PE-labeled pentamers comprising HLA-A^*^02:01-bound HCV NS3_1406−1415_ (KLSALGINAV) and HLA-A^*^01:01-bound HCV NS3_1435−1443_ (ATDALMTGY) were used to stain HCV NSmut-specific CD8+ T cells (Proimmune). The specificity of pentamers was confirmed on HLA-matched pre-vaccination samples from healthy individuals (31). Thawed PBMC (1–2 × 10^6^) were coincubated with pentamers and antibodies specific for cell surface or intracellular proteins, as listed in Supplementary Table 2). Stained samples were fixed using 1% paraformaldehyde and, where applicable, permeabilised using 10 × permeabilisation buffer (eBioscience). Data were collected with BD FACS DIVA software (San Jose, CA, USA) and analyzed with Flow Jo software (Flowjo LLC, Ashland, OR, USA).

### HLA Typing

HLA typing was performed by amplification refractory mutation system (ARMS) PCR using sequence-specific primers.

### Chimpanzee Adenovirus Neutralization Assay

ChAd3 and ChAd63 neutralizing antibody (NAb) titres were assayed as previously described using a secreted alkaline phosphatase (SEAP) assay (35). Briefly, 8 × 10^4^ HEK293 cells per well were seeded in a 96-well-plate for 1 day. SEAP-expressing ChAds were pre-incubated for 1 h at 37°C alone or with serial dilutions of heat-inactivated serum from trial volunteers, added to the 80–90% confluent HEK293 cells for 1 h at 37°C, after which the supernatant was replaced with 10% FBS in DMEM. SEAP activity in the supernatant was measured after 24 ± 2 h using the chemiluminescent substrate (CSPD) from Phospha-Light kit (Tropix) following the manufacturer's instructions. Light signal output expressed as relative light units (RLU) was measured 45 min after the addition of the CSPD substrate using a luminometer (Envision 2102 Multilabel reader, Perkin Elmer). The neutralization titer was defined as the reciprocal of sera dilution required to inhibit SEAP expression by 50% compared to the SEAP expression of virus infection alone. The lowest dilution tested was 1:18, therefore, a neutralization titer of < 1:18 was used as the negative cut-off.

### RNA Isolation and Microarray

Blood samples for transcriptomic analysis of the innate immune response were collected in Paxgene^®;^ tubes at weeks 0 (days 0 and 1), 7 and 8 (days 56 and 57). RNA was isolated using the PAXgene Blood RNA kit (Qiagen) according to the manufacturer's protocol. Following globin depletion (GLOBINclear™ Kit, ThermoFisher SCIENTIFIC) total RNA was quantified using a NanoDrop spectrophotometer. Transcription amplification and labeling (Illumina^®;^ TotalPrep™-96 RNA Amplification Kit, Ambion) was performed starting with 50 ng RNA per sample. After hybridization (Whole-Genome Gene Expression Direct Hybridization, Illumina), the slides were scanned on the Illumina iScan system (Wellcome Trust Centre for Human Genetics, University of Oxford).

### Host Transcriptome Analysis

The raw Illumina microarray probe data were annotated into probe sets representing individual genes, using readBeadSummaryData. Outliers were eliminated from the data set and gene expression data were normalized using the NEQC function (36). Two volunteers from Group 2 and one volunteer from Group 3 were subsequently eliminated. The array weight was calculated (Limma arrayWeight) to define the minimal matrix to reflect the experimental settings (pre- and post-vaccination time points). The probes that had *p*-value < 0.05 in at least 10% of the samples were filtered. Gene expression values (log_2_) at time points post-vaccination (day 1 and week 8 + 1 day) were converted to fold changes by subtracting the respective pre-vaccination (days 0 and 56) expression values for the same genes. Log_2_ expression values were tested for statistical significance by ANOVA adjusted to false discovery rates using the Benjamini–Hochberg False Discovery algorithm. Genes were defined as differentially expressed post-vaccination if the fold ratio was ≥1.5 and *p*-value ≤ 0.001. Gene networks and their functional interactions were analyzed with Gene Ontology (GO) enrichment analysis and visualization tool (http://cbl-gorilla.cs.technion.ac.il/), STRING (https://string-db.org) and RStudio. Group 1 samples were not analyzed for this study because a similar analysis had been performed previously on 6 healthy volunteers who had received the same vaccine regimen (ChA3-NSmut 2.5 × 10^10^ vp on day 0 followed by MVA-NSmut (2 × 10^8^ pfu) on day 56 (week 8) and bleed schedule in the HCV003 trial (NCT01296451 (L. Swadling, personal communication). Instead, we made a comparison of the data from Group 2 and 3 subjects with corresponding whole blood transcriptomic data from the HCV003 subjects, as this had been generated using the same methods.

### Safety and Immunological Data Analysis

Safety and clinical laboratory data were entered on OpenClinica software and checked by two staff members. For continuous variables, the mean with standard deviation was used to summarize hematological parameters; median with IQR were used to summarize the immunogenicity data. Differences in hematological parameters over time were tested for significance by 1-way ANOVA. Differences between groups in the magnitude and breadth of ELISpot responses over time were tested by multiple *t*-tests with correction for multiple comparisons using the Holm-Sidak method (alpha = 0.05). Other analyses were performed using non-parametric tests. Statistical analysis of safety and immunological data was performed using GraphPad Prism version 7.

## Results

### Co-administration of Serologically Distant Adenoviral Vectors Is Safe and Well-Tolerated

**Figure 1:**
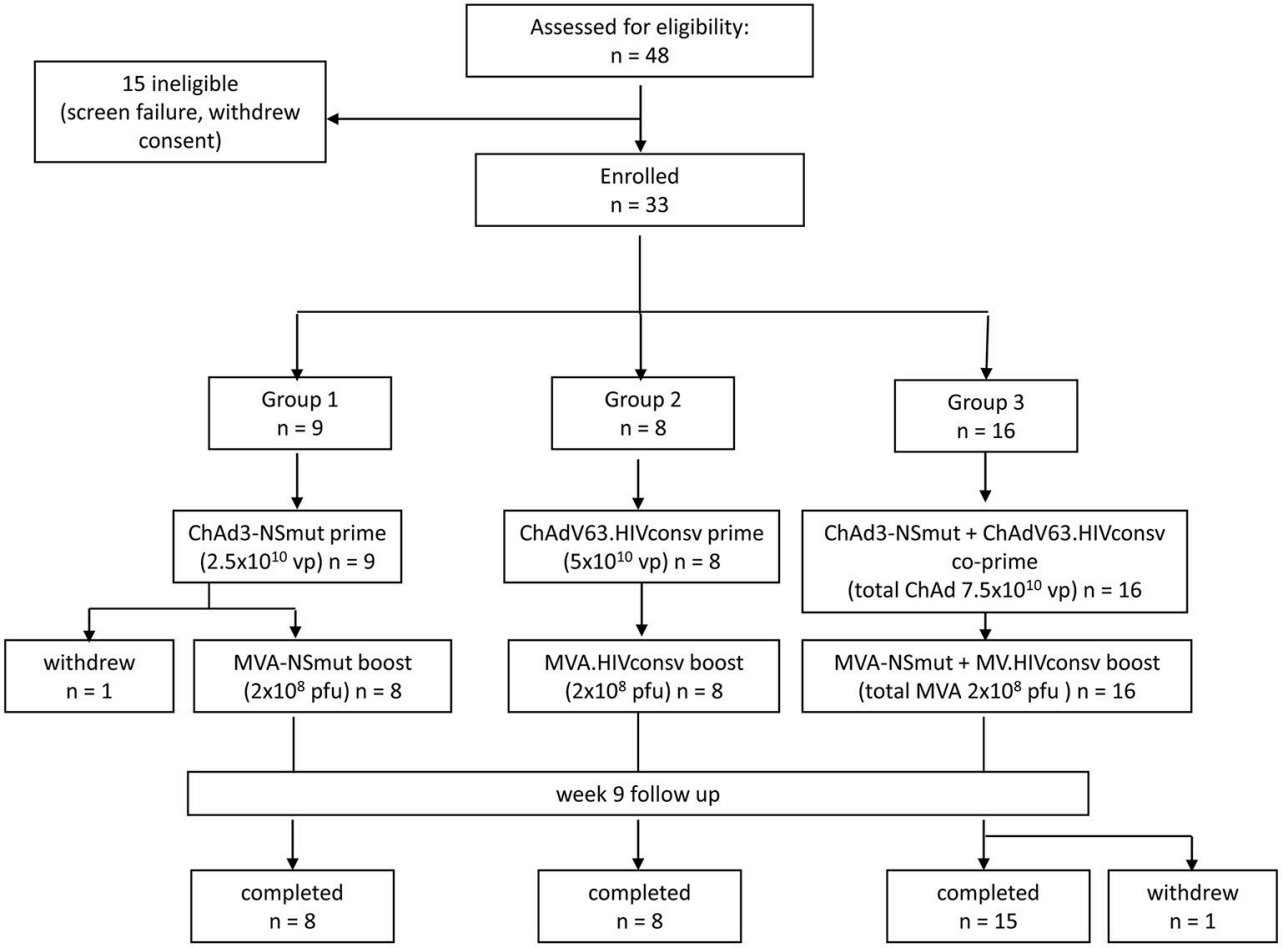
Consort flow diagram showing enrolment and follow-up in the PEACHI 04 trial.

Forty-eight subjects were screened for eligibility and 33 were enrolled (19 females and 14 males). Vaccine allocation is shown in Figure 1. Mean age at enrolment was 29 years (range 19–48 years). One volunteer in Group 1 withdrew consent after receiving the ChAd3-NSmut vaccination and was replaced. All other volunteers completed the immunization schedule. One volunteer in Group 3 withdrew consent after receiving all vaccinations (week 12) but before completion of follow-up (Participant flow chart, Figure 1). There were no suspected unexpected serious adverse reactions or serious adverse events. There were 301 solicited local and systemic adverse events in total: 137 after priming vaccinations and 164 after boosting vaccinations. Overall, the majority of events related to vaccination were mild (grade 1) in severity (178/301, 59%) and resolved within 48 h.

**Figure 2:**
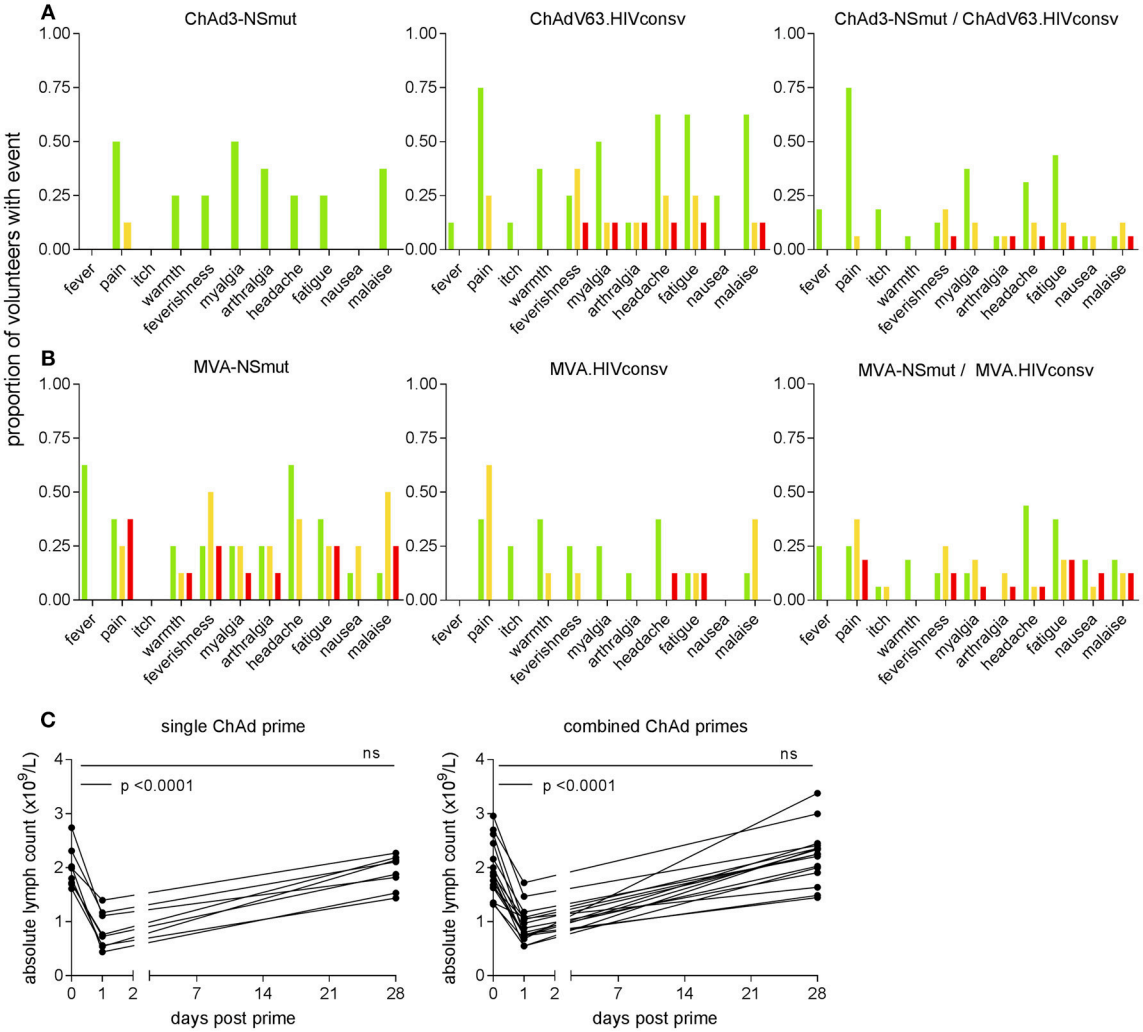
Frequency of local and systemic adverse events recorded by volunteers on diary cards. The proportion of volunteers reporting symptoms at any time during 72 h following **(A)** ChAd- and **(B)** MVA-vectored vaccines is shown on the y axis. Color code indicates maximum severity of the reaction reported: green—Grade 1 (mild); yellow—Grade 2 (moderate); red—Grade 3 (severe). **(C)** Peripheral blood lymphocyte counts were measured on day 1 in 23 subjects (two in Group 1 and five in Group 2, shown as single ChAd prime; 16 in Group 3, combined ChAd primes). Respective lymphocyte counts on days 0 and 28 are shown for comparison. By day 28, counts had returned to baseline values.

The most frequently reported solicited events were pain at the injection site, myalgia, fatigue, headache and malaise. ChAd-vectored vaccines were less reactogenic (38/137, 28% grade 2 or3) than MVA vectored vaccines (85/164, 52% grade 2 or 3) (Figures 2A,B). ChAdV63.HIVconsv appeared to be more reactogenic than ChAd3-NSmut, with four volunteers in Group 2 reporting at least one symptom of grade 2 or 3 compared with only one in Group 1, possibly reflecting the different doses of viral vector that were administered. Co-administration of the two MVA-vectored vaccines at half the dose used in the single regimen groups was as well-tolerated as vaccination with either alone, likely reflecting the equivalent total dose of recombinant MVAs across the three groups (Figures 2A,B).

The only abnormal laboratory parameter of note was a transient fall in total lymphocyte count within 24 h of administration of ChAd3-NSmut and ChAdV63.HIVconsv vaccines. This became apparent in 24 volunteers after implementation of a protocol amendment to perform a full blood count estimation on day 1 post-vaccination as an additional safety evaluation. Three episodes were classified as grade 1 (0.8–0.91 × 10^9^ cells/L), 11 were grade 2 (0.5–0.8 × 10^9^ cells/L), one was grade 3 (0.2–0.5 × 10^9^ cells/L); 9 did not meet grading criteria. Mean (SD) counts (x10^9^ cells/L) at day 0 and 1 were 1.99 (0.4) and 0.84 (0.3) for volunteers who received a single vaccine regimen and 1.95 (0.5) and 0.94 (0.3) for volunteers given the combined vaccine regimen (*p* < 0.0001 for both, Figure 2C). These changes were not associated with any symptoms and lymphocyte counts returned to normal by week 4 in all cases. Other hematological parameters did not change significantly between days 0 and 1, except for a slight increase in monocyte counts (×10^9^ cells/L) from a mean (SD) of 0.52 (0.2) to 0.7 (0.2) in the combined vaccination arm (*p* = 0.01). Unsolicited adverse events including abnormal laboratory parameters are listed in Supplementary Tables 3, 4.

### Magnitude and Breadth of Transgene Product-Specific T Cell Responses Induced by Combined Vaccine Regimens Are Similar to Those of Each When Given Alone

*Ex vivo* IFN-γ ELISpot assays were performed in all volunteers at weeks 0, 1, 2, 4, 8, 9, 12, 14, and 34. One subject from each of Groups 2 and 3 was excluded from the ELISpot analysis due to mock-stimulated values exceeding the assay cut-off at three or more time-points.

**Figure 3:**
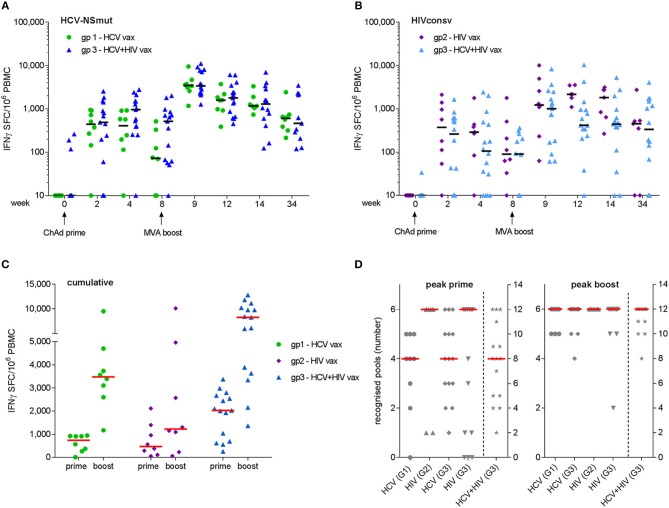
Frequency of antigen-specific T cells as determined by fresh *ex vivo* IFN-γ ELISpot assays. Mock-subtracted values are shown. Summed responses to peptide pools spanning **(A)** NSmut in Group 1 (green) and Group 3 (blue) volunteers and **(B)** HIVconsv in Group 2 (purple) and Group 3 (light blue) volunteers. Black horizontal bars indicate median values. **(C)** Cumulative responses (sum of NSmut and HIVconsv) in Group 3 subjects are compared with responses to the same immunogens when administered as a single regimen. **(D)** Breadth (number of peptide pools eliciting a positive response) at the time of the peak response to priming and boosting vaccinations. Group 3 (G3) values in right hand columns indicate the sum of HCV and HIV pools recognized. For **(C,D)**, red bars indicate median values.

Peak responses to ChAd3-NSmut/MVA-NSmut were observed at week 9 in 6/8 volunteers in Group 1 and 14/14 volunteers in Group 3. Median (IQR) responses at week 9 were 3,480 (2,728–4,464) and 3,405 (2,307–7,804) SFC/10^6^ PBMC for Groups 1 and 3, respectively (Figure 3A). Peak responses to ChAdV63.HIVconsv/MVA.HIVconsv were detected at week 9 in 5/7 volunteers in Group 2 and 9/14 volunteers in Group 3. Median (IQR) responses at week 9 were 1,305 (1,095–4,967) and 1,005 (169–2,482) SFC/10^6^ PBMC for Groups 2 and 3, respectively (Figure 3B). Responses at the final visit (week 34) remained elevated above baseline in all groups (NSmut, Groups 1 and 3—*p* = 0.008 and 0.002, respectively; HIVconsv, Groups 2 and 3—*p* = 0.06 and 0.001, respectively, Wilcoxon matched pairs test). Co-administration of HCV and HIV vaccines did not have a significant impact on the magnitude of responses to NSmut (Group 1 vs. Group 3) or HIVconsv (Group 2 vs. Group 3) at any time point (FDR, *Q* = 1%) (Figures 3A,B). Cumulative responses in Group 3 (sum of NSmut- and HIVconsv-specific T cells) exceeded responses to either immunogen alone after boosting vaccinations (multiple *t*-tests: *p* = 0.008 and 0.0006 for NSmut and HIVconsv, respectively). Taken together, these data indicate that there was no immune interference when the vaccines were co-administered. Furthermore, all vaccinees responded to transgene products at one or more time points during follow-up (Figure 3C).

The minimum breadth of responses to each immunogen was determined from the number of peptide pools that gave a positive response, as defined in Materials and Methods. After priming vaccinations, the median breadth was 4/6 NSmut pools for both Groups 1 and 3, 6/6 HIVconsv pools for Groups 2 and 4/6 HIVconsv pools for Group 3. After boosting vaccinations, the median breadth was 6/6 pools for all groups, with most volunteers in Group 3 developing T cell responses to all 12 HCV and HIV peptide pools (Figure 3D). Cumulative breadth in Group 3 (sum of NSmut and HIVconsv pools recognized) also exceeded the breadth of responses in Groups 1 and 2 after boosting vaccinations (multiple *t*-tests: *p* < 0.0001 for both), confirming that co-administration of the HCV and HIV vaccines in prime-boost regimens achieved similarly broad responses to those observed after single vaccinations. It should be noted that this may be a conservative estimate of the response breadth since the response to any given pool may have been targeted to more than one epitope.

### Specificity and Immunodominance of Vaccine-Induced T Cell Responses Is Maintained When the HCV and HIV Vaccines Are Co-administered

**Figure 4:**
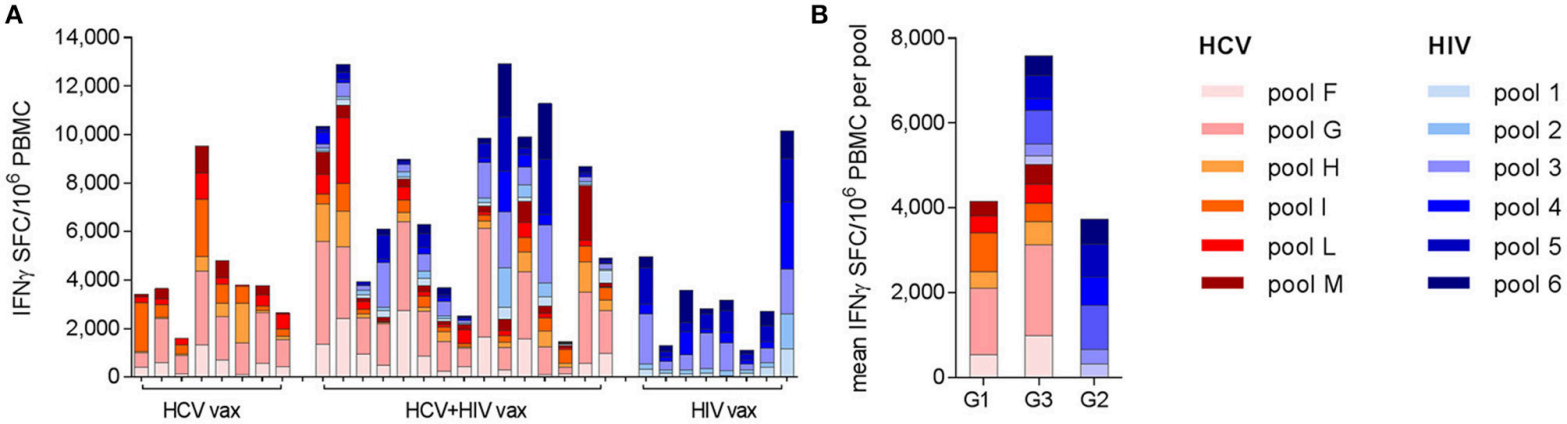
Breadth and specificity of antigen-specific T cell responses as determined by *ex vivo* IFN-γ ELISpot assays: each bar shows the contribution of individual peptide pools to the total response to the NSmut (red shades) and HIVconsv (blue shades) immunogens at the peak of the response after boosting vaccinations. Although the peak response was most frequently observed at day 63, the day 84 or 98 response is shown for the few individuals with a later peak, to illustrate the maximal breadth of individual responses. **(A)** Individual subjects are shown, indicated by trial identifier on x axis; **(B)** mean values per group.

Analysis of the targets of vaccine-induced T cells showed that responses to both immunogens were broad but were dominated by HCV NS3 helicase (pool G) and HIV-1 Pol (pool 3), after priming and boosting vaccinations with single regimens. These immunodominance hierarchies were maintained when HCV and HIV vaccines were co-administered, irrespective of whether the responses in Group 3 subjects were HCV-dominant (12/15 subjects) or HIV-dominant (3/15 subjects) (Figure 4). Although responses were not mapped to individual peptides in this trial, the immune response hierarchies were consistent with the genetic background of the volunteers: HLA-A^*^02 alleles were highly represented in all groups (4/9 in Group 1, 5/8 in Group 2 and 10/16 in Group 3; several HLA-A^*^02-restricted epitopes have previously been identified in both NS3 pool G and HIVconsv pool 3 (37, 38).

### Induction of Multi-Functional Antigen-Specific CD4^+^ and CD8^+^ T Cells With Single and Combined Vaccine Regimens

**Figure 5:**
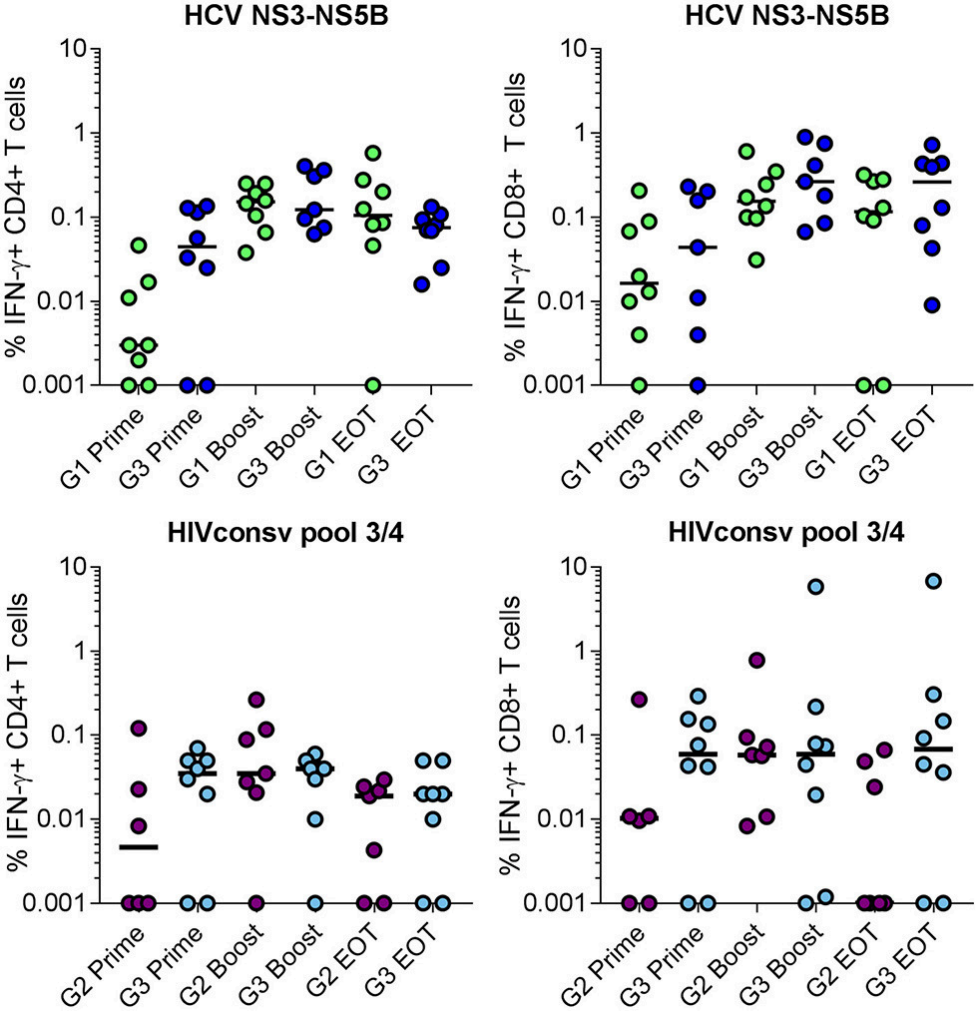
Frequency of total antigen-specific IFN-γ^+^ cells within (left) CD4^+^ and (right) CD8^+^ T populations in volunteers in Groups 1 and 3 (NSmut vaccines, top panels) and Groups 2 and 3 (HIVconsv vaccines, bottom panels) after priming, boosting and at the end of trial (EOT) as determined by intracellular cytokine staining of cryopreserved PBMC. Zero values were arbitrarily assigned a value of 0.001 to enable display on a log_10_ scale. Horizontal lines indicate median values. Groups were compared using Kruskall-Wallis test; no statistically significant differences were found.

The functional capacity of antigen-specific CD4^+^ and CD8^+^ T cell populations was analyzed in all volunteers after priming, boosting and at the end of the trial. The gating strategy used to identify antigen-specific T cells is shown in Supplementary Figure 2. As responses were dominated by IFN-γ production, comparisons between single and combined vaccine regimens in the frequencies of IFN-γ-producing cells in each subset are shown (Figure 5). The median NSmut-specific responses post-boost were similar for Groups 1 and 3 and were 0.15 and 0.12%, respectively for CD4^+^ T cells and 0.15 and 0.26%, respectively for CD8^+^ T cells (Figure 5; Supplementary Table 5). The median HIVconsv-specific responses post-boost were also comparable for Groups 2 and 3 and were 0.035 and 0.04%, respectively for CD4^+^ T cells and 0.06 and 0.06%, respectively for CD8^+^ T cells (Figure 5; Supplementary Table 6). Overall, the frequencies of antigen-specific responses detected by intracellular staining for IFN-γ were lower than predicted by the ELISpot assay data, which may reflect differences in the assay sensitivities (39).

These observations extended to the frequencies of other cytokine-producing populations in the single and combined vaccination groups (Supplementary Tables 5, 6). The dominant antigen-specific marker expressed by CD8^+^ T cells after IFN-γ was CD107a: median peak frequencies of NSmut-specific cells in Groups 1 and 3 were 0.19 and 0.17%, respectively; corresponding frequencies of HIVconsv-specific cells in Groups 2 and 3 were 0.07 and 0.02%, respectively. The dominant signal in CD4^+^ T cells after IFN-γ was CD154 (CD40 ligand, which is upregulated on activated CD4^+^ T cells), with median peak frequencies of NSmut-specific cells in Groups 1 and 3 of 0.15 and 0.18%, respectively and of HIVconsv-specific cells in Groups 2 and 3 of 0.06 and 0.05%, respectively. In all groups, we detected transgene product-specific CD4+ T cells that co-expressed IFN-γ, CD154, TNF-α and IL-2 and CD8^+^ T cells that co-expressed IFN-γ, CD107a, and TNF-α (SPICE analysis, Supplementary Figure 3).

The phenotype of NSmut-specific CD8^+^ T cells was investigated further using HLA-A^*^02:01- and HLA-A^*^01:01-peptide pentamers. The median frequencies of pentamer-positive cells after ChAd3-NSmut priming were 0.014% and 0.49% CD8^+^ T cells for Groups 1 and 3, respectively. These increased to 3.78 and 4.4%, respectively after the MVA-NSmut boost (week 9) and were still detectable at the end of the trial (0.27 and 0.42%) (Supplementary Figures 4A,B). Pentamer-positive populations were dominated by effector memory T cells (T_EM_, CD45RA- CCR7-) that were highly activated (CD38^+^/HLA-DR+) following prime and boost vaccinations, whereas by the end of the trial, a substantial proportion of these had been replaced by terminally differentiated effectors (T_EMRA_, CD45RA^+^ CCR7^−^) (Supplementary Figure 4C). The vast majority of pentamer-positive cells expressed granzymes A and B, apart from at the end of trial, when frequencies of Granzyme A-positive cells were significantly lower in Group 3 than Group 1 (Supplementary Figures 4D,E).

Collectively, these results indicate that the function and phenotype of vaccine-induced HCV- and HIV-specific T cell populations were maintained when the respective vaccines were co-administered.

### Adenovirus Type-Specific Neutralizing Activities Were not Affected by Adenoviral Vector Co-administration

**Figure 6:**
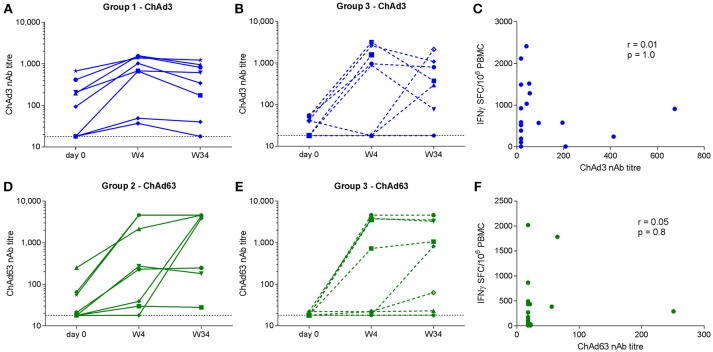
Neutralizing antibody titres to ChAd3 **(A,B)** and ChAd63 **(D,E)** measured in serum on day 0, week 4, and week 34 (end of trial) in volunteers receiving single (left) and combined (middle) vaccination regimens. Horizontal dotted line indicates assay cut-off (titer of 18). Correlation analysis (Spearman) for pre-vaccination vector-specific nAb titres and magnitude of the respective total transgene-specific T cell responses at W4 are shown in **(C,F)**.

ChAd-specific nAb titres were measured pre-vaccination and at weeks 4 and 34 in all Group 1 and Group 2 subjects and 10/16 Group 3 subjects. At baseline, neutralizing activity against ChAd3 and ChAd63 was detectable in 9/18 and 6/18 subjects, respectively, although titres were < 200 in the majority. An increase in ChAd3 or ChAd63 titer of at least 4-fold was observed in 8/18 Group 1 and 3 subjects and in 10/18 Group 2 and 3 subjects by week 4 (Figures 6A–D). Geometric mean values for ChAd3-specific nAbs were 479 and 171 for Groups 1 and 3, respectively (*p* = 0.9). Geometric mean values for ChAd63-specific nAbs were 298 and 234 for Groups 2 and 3, respectively (*p* = 0.9). Although we could not discern a clear relationship between baseline and post-vaccination neutralization titres due to the small numbers of subjects analyzed these results suggest that co-administration did not affect the nAb titer to the reciprocal vector at peak or 8 months after priming.

We also investigated whether pre-existing ChAd-specific humoral immunity impacted on priming of transgene-specific T cell responses. No correlations between the baseline ChAd-specific nAb titer and the magnitude of the respective T cell response post-prime were observed (ChAd3 nAb vs. week 4 NSmut SFC/10^6^ PBMC, *r* = 0.01, *p* = 1.0; ChAd63 nAb vs. week 4 HIVconsv SFC/10^6^ PBMC, *r* = 0.05, *p* = 0.8) (Figures 6E,F), suggesting that pre-existing nAbs at the titres observed here did not significantly inhibit vector uptake into target cells.

### Whole Blood Transcriptome Signatures 24 h Post-vaccination Are Similar for Single and Combined Vaccine Groups

**Figure 7:**
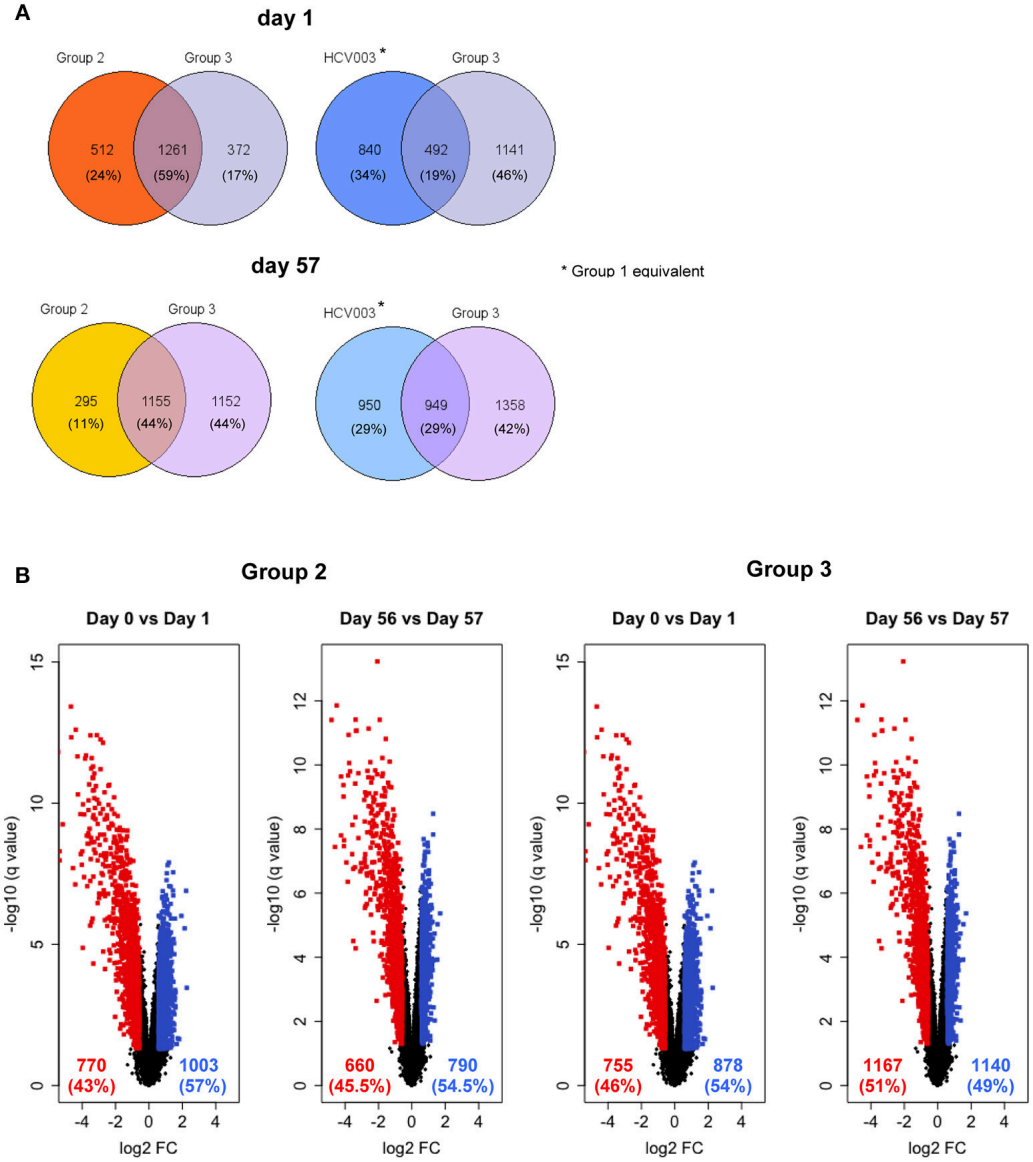
**(A)** Venn diagrams showing the proportion of genes that were significantly differentially expressed on days 1 and 57, i.e., 24 h post-prime and post-boost vaccination, respectively, with data from the HCV003 trial participants (ChAd3-NSmut/MVA-NSmut, L. Swadling, personal communication) included for comparison with Groups 2 and 3. **(B)** Volcano plots illustrate the numbers of the differentially expressed genes 24 h after vaccination. Red dots—genes significantly downregulated; blue dots—significantly upregulated).

Innate gene expression after vaccination has been shown to determine functional adaptive immune responses, therefore, an unbiased analysis of whole blood transcriptomes was undertaken to investigate this (40–42). The global view of gene expression analysis in total blood cells following each vaccine administration showed that days 1 and 57 clustered together in both Groups 2 and 3 (Supplementary Figure 5). Comparison of the gene expression profiles on day 1 with day 0 revealed that 1,773 and 1,633 genes were differentially expressed in Groups 2 and 3, respectively, of which 1,261 genes (59%) were common to these groups (Figure 7A). Group 1 subjects were not analyzed as subjects in the HCV003 trial received the same vaccination regimen (see Methods; L. Swadling, personal communication) and had already been subjected to the same transcriptional analysis. This showed that 1,332 genes were differentially expressed, of which 492 (19%) were shared with Group 3 (Figure 7A). Gene expression profiles were also substantially affected by booster vaccinations, with differential expression of 1,450 and 2,307 genes in Groups 2 and 3, of which 1,155 (44%) were shared between Groups 2 and 3. In HCV003 participants, 1,899 genes were differentially expressed after boosting, of which 949 (21%) were shared with Group 3 (Figure 7A). The number of differentially expressed genes (DEGs) stratified by up- and down-regulation after priming and boosting vaccinations is also shown for Groups 2 and 3 as volcano plots (Figure 7B). Comparison of Groups 2 and 3 showed that the proportions of both down- and upregulated genes were similar after priming and also after boosting.

**Figure 8:**
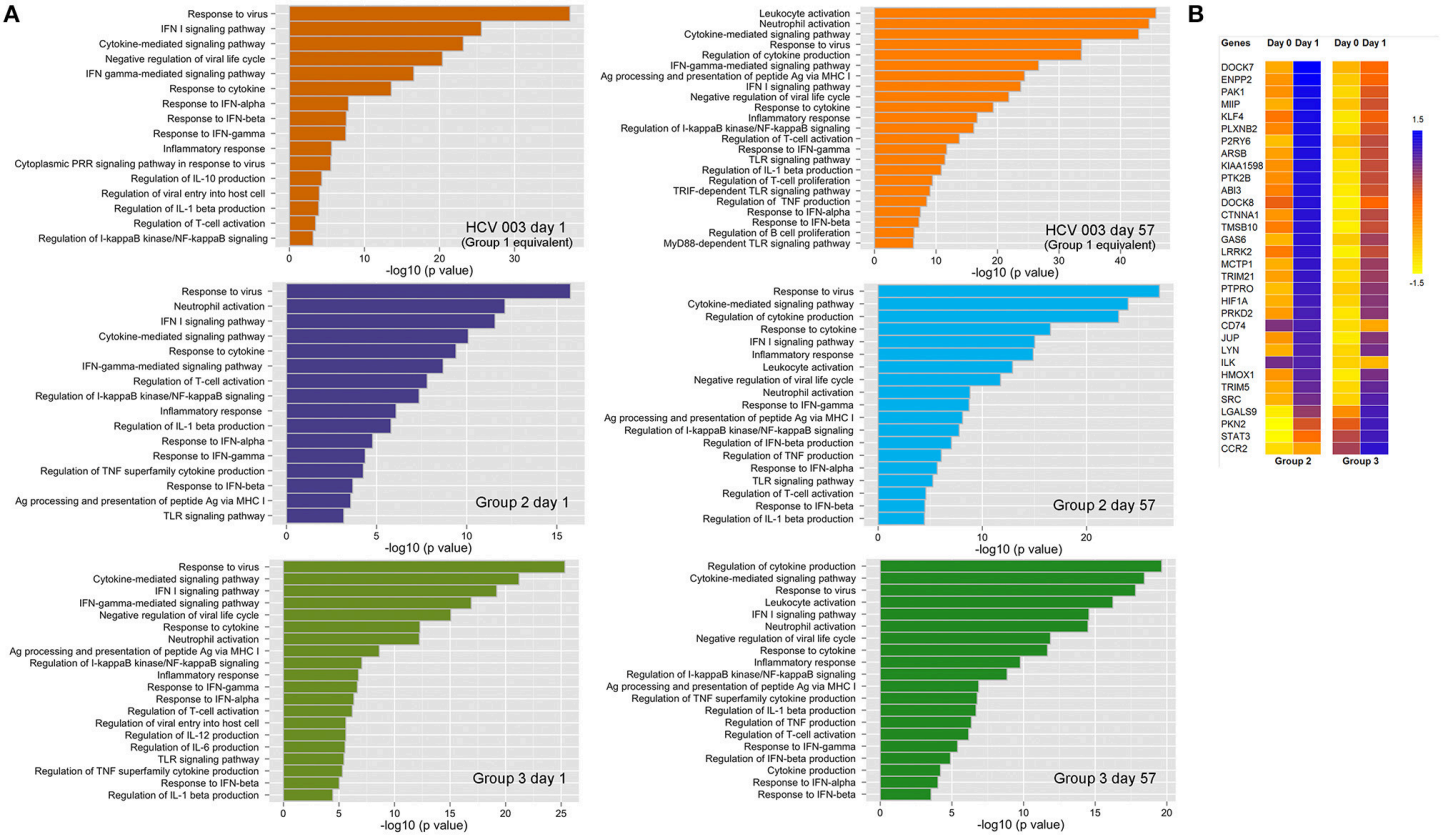
**(A)** Canonical pathway analysis modulated by differentially expressed genes (DEGs) that were upregulated 24 h post each vaccine administration: left panels—ChAd-vectored vaccines; right panels—MVA-vectored vaccines. The biological processes are depicted from DEGs in whole blood. The pathways are indicated on the y axis, and the x axis shows the significance score (negative log_10_ of *p*-value calculated using Fisher exact test). **(B)** Heatmap depicting expression of genes involved in locomotion (GO: 0040012), which are correlated with lymphocyte count decline on day 1. Groups 2 and 3 are shown separately but were pooled for statistical analysis as the change in lymphocyte count was similar in the two groups.

Canonical pathway analysis was used to identify pathways that are enriched within the set of DEGs that were significantly upregulated. In the three vaccine groups the major pathways included the antiviral response, IFN-I and IFN-II signaling and response, regulation of IL-1-β secretion, inflammatory responses, negative regulation of viral life cycle and regulation of T-cell activation, after both priming and boosting vaccinations (Figure 8A; Supplementary Table 7). However, some differences were observed with each vaccine regimen: 24 h post-priming, pathways such as neutrophil and leukocyte activation, TLR signaling and regulation of IL-6 and IL-12 were only enriched in ChAd63 (Group 2) and combined ChAd3/ChAd63 vaccine recipients (Group 3), whereas the cytoplasmic pattern recognition receptor signaling and regulation of IL-10 pathways were only enriched in HCV003 vaccinees (Figure 8A). After boosting vaccinations, this group also showed enrichment of TRIF– and MyD88-dependent TLR signaling pathways (Figure 8; Supplementary Table 7).

In view of the transient fall in peripheral blood lymphocyte counts following administration of ChAd-vectored vaccines we examined the association between lymphocyte count reduction and DEGs. Groups 2 and 3 were combined since there were no significant between-group differences in lymphocyte counts pre- and post-vaccination (unpaired *t*-test for day 0—*p* = 0.9; day 1—*p* = 0.5). This analysis revealed that a total 289 of the upregulated genes were correlated with the observed lymphopenia. Thirty-one of these genes were found to be associated with regulation of locomotion (GO:0040012; Figure 8B). Furthermore, 329 of downregulated genes were correlated with lymphopenia post-ChAd vaccination. Many of these are involved with biological functions other than the immune response (e.g., initiation of RNA translation, elongation and termination), while only 16 are associated with the immune response, e.g., regulation of lymphocyte activation (data not shown).

## Discussion

This trial has demonstrated that co-administration of vaccine candidates comprising serologically distant simian adenoviruses and MVA vectors that each encoded sequences from HCV and HIV-1 was safe, well-tolerated and did not impair the immunogenicity of either vaccine regimen.

The frequency and severity of local and systemic reactions following dual immunizations with either ChAd- or MVA-vectored vaccines were similar to those observed following administration of each vaccine regimen alone. The higher frequency of moderate or severe reactions after priming with ChAdV63.HIVconsv, whether alone or in combination with ChAd3-NSmut, could reflect the higher doses of the ChAd63 vector that were used (43, 44). The dose of each MVA vectored vaccine, on the other hand, was halved for the co-administration arm to avoid unacceptable reactogenicity. Overall, the safety and tolerability profile of the four vaccines was similar to that reported previously (32, 34).

As adenovirus-vectored vaccines targeting several globally distributed pathogens advance to phase 2/3 trials in overlapping populations, the possibility of anti-vector immunity and antigenic competition, which may each adversely affect the efficacy of existing immunization programmes and competing vaccine candidates, is a legitimate concern. We have shown that the magnitude, breadth, durability and quality of T cell responses to the HCV and HIV immunogens were not significantly affected by co-administration, and that responses measured in this trial were similar to those reported in previous trials in all these respects. There was, however, a trend toward lower magnitude of HIVconsv-specific T cell responses when given in combination with the HCV vaccines. The optimal boosting dose of MVA.HIVconsv has not been defined and we cannot exclude the possibility that the lower dose of MVA.HIVconsv tested in Group 3 was suboptimal.

We did not find evidence that transgene-specific T cell responses were impacted by pre-existing anti-vector immunity as the majority of study participants had ChAd-specific nAb titres below the level of detection, possibly reflecting the age of the study population (45, 46). However, vector-specific nAbs were induced or boosted in the majority of vaccinees by week 4 and were maintained to week 34 at titres >200. Given that adenovirus-specific nAbs were shown to dampen responses to a heterologous Ad boost when these were administered after a 20 week interval (31), our results support the notion that administration of serologically related Ad in combination rather than sequentially is a useful strategy to minimize the potential for unwanted effects of anti-vector immunity.

This study enabled a direct comparison of responses to the HCV and HIV vaccine regimens, which revealed differences in the magnitude of induced T cell responses. NSmut-specific T cell frequencies were generally of higher magnitude. Various mechanisms may be implicated. ChAd3 was found to be more potent than ChAd63 (and also rare human adenovirus serotypes) for induction of SIV-specific CD8^+^ T cells in a mouse model, therefore, it may be expected that differences in T cell priming efficiency would be evident in humans (47). More plausibly, the NSmut and HIVconsv immunogens differed considerably in length (1,985 and 777 aa, respectively) and this is likely to be a determinant of the number of potential T cell epitopes. Although the breadth of induced T cell responses was not precisely defined, the NS3-5 region of the HCV proteome is known to contain at least 12 epitopes that are restricted by HLA-A^*^02 alleles, which were highly represented in the study population, along with many more that are restricted by other class I and HLA class II alleles, in particular, HLA-DR-restricted T cell epitopes that are frequently recognized by people who spontaneously resolved HCV infection (48–50). Furthermore, many of these epitopes are immunodominant. The HIVconsv immunogen, on the other hand, was specifically designed to minimize the targeting of immunodominant regions, or “hotspots,” in which viral escape frequently occurs, as was observed with native protein sequences in Ad5-vectored vaccines (51, 52). Instead, the aim was to direct responses to regions that are subdominant in natural infection. A mapping study in healthy volunteers in the first HIVconsv vaccine trial confirmed this: while responses to at least two known HLA-A^*^02-restricted Pol epitopes in 3/12 subjects were observed, many new epitopes in HIVconsv were identified, yet with a high degree of heterogeneity in responses across the study population (38).

Multiparameter flow cytometric analysis indicated that responses to both the NSmut and HIVconsv immunogens were balanced with respect to the contribution of CD4^+^ and CD8^+^ T cells and that these comprised sub-populations expressing combinations of IFN-γ, TNF-α, IL-2, CD107a, and CD154. This was more evident in the HCV vaccine recipients due to the higher magnitude of NSmut-specific T cell responses. Nonetheless, the functional profiles observed in both HCV and HIV vaccinees were similar in the single and combined vaccine groups and were consistent with data from previous trials of these vaccines (32, 33). The cytokine profile of CD4^+^ and CD8^+^ T cells was reflective of a state of cleared or limited viral antigen expression, which has been described in other healthy vaccinated populations and in HIV controllers (32, 33, 50, 53, 54). Furthermore, pentamer staining showed a similar kinetic of T cell activation and expansion of NSmut-specific CD8^+^ T cells to that described following administration of yellow fever, smallpox and other recombinant MVA vaccines (55, 56). Interestingly, although the majority of pentamer–positive populations induced following prime and boost vaccinations were of a T_EM_ phenotype, these had had contracted by the end of the trial, as would be expected in the context of vaccination or infection with a non-persistent virus; however, a majority of these cells had switched to a T_EMRA_ phenotype with high levels of granzymes being expressed. This is suggestive of ongoing antigen expression, which has been reported previously in the context of replication-deficient adenovirus vectors and was attributed to persistence of transcriptionally active viral genomes at the site of immunization and in secondary lymphoid organs (57, 58).

We observed transient lymphopenia 24 h post-administration of ChAd-vectored vaccines, which was of similar severity in those receiving single or combined vaccinations. This was likely due to migration to secondary lymphoid tissues, as the whole blood transcriptome showed significant up- and down-regulation of genes involved in locomotion and regulation of lymphocyte activation, respectively. Although we did not resolve the lymphocyte subset changes, preliminary data indicate that a reduction in absolute numbers of NK cells occurred during the first 24 h (C. M. Gardiner, personal communication). Transient lymphopenia has been reported previously in a clinical trial of a ChAd63-vectored Leishmania vaccine; however, in this study it was attributed to a predominant reduction in the frequency of CD4^+^ T cells (59). We also observed that lymphopenia was temporally associated with the upregulation of CCR2, LYN, PAK1, PTK2B, SRC, STAT3, which are involved in the chemokine signaling pathway. This may have accounted for the concomitant increase in monocyte counts in the peripheral blood, which was more pronounced in Group 3 subjects. At the same time, genes involved in inflammation and interferon signaling were significantly upregulated, as has been reported in other vaccine studies including those involving adenovirus-vectored vaccines (42, 59). The observed changes were broadly similar in Groups 2 and 3 and HCV003 (equivalent to Group 1), suggesting that dual administration of adenovirus vectors did not amplify the effects of either alone. However, the antigen processing and presentation via MHC class I pathway was upregulated in Groups 2 and 3 and not in HCV003 subjects. Furthermore, many of the genes involved in the pattern recognition receptor signaling pathway (e.g., *MYD88, CD36, CD 86, TANK*, and *TLR8*), and in the T cell receptor signaling pathway *(HLA-DRA, HLA-DQB1, HLA-DRB3, HLA-DPA1*, and *HLA-DB1)* were not differentially expressed following ChAd3-NSmut vaccination. These observations suggest that there are some subtle differences in the ChAd3 and ChAd63 vectors and/or the design of the inserts that may impact on the quality of immune responses to encoded antigens, although no substantial differences in magnitude or functionality were evident from the assays performed in this trial. The transcriptomic signatures induced by the ChAd-vectored vaccines showed some overlap with the effects of the MVA-vectored vaccines, with response to virus, type I interferon signaling and cytokine-mediated signaling pathways being highly represented in all groups. In addition, profound upregulation of genes involved in leukocyte activation and the inflammatory response was observed, in common with previously described effects of other MVA-vectored vaccines (60). Limitations of this analysis were the use of whole blood, which does not take into account variation in frequency of specific cell populations. However, these data provide a foundation for further dissection of heterogeneity in the host response to vaccine candidates that may enable the identification of biomarkers of efficacy.

In conclusion, we have demonstrated the feasibility of co-administering two viral vector vaccine regimens to healthy human subjects without undermining safety, tolerability or induction of transgene product-specific T cell responses. Given that there is an unmet need for effective vaccines not only for control of established pandemics caused by HCV and HIV but also for emerging and outbreak pathogens, combination approaches will be crucial for ensuring maximal adherence and population coverage and for minimizing the impact of anti-vector immunity.

## Author Contributions

SC, AN, AF, TH, EB, and LD: study design; FH, PC, and PM: conducted clinical trial; FH, AB, SC, JK, CBl, SM-N, LS, EG, MD, RS, and IE: conducted experiments; FH, AB, SC, JK, CBl, SM-N, LS, EG, PC, MD, RS, IE, BT, EB, and LD: analyzed data; FH, SC, SM-N, LS, EB, and LD: wrote manuscript; SC, VV, CG, CBa, CBe, MH, BT, AF, TH, EB, and LD: project management and supervision.

### Conflict of Interest Statement

VV is an employee of GSK and owns restricted shares of the company. AN and AF are named inventors on patent applications covering HCV-vectored vaccines and chimpanzee adenovirus vectors [WO 2006133911 (A3) hepatitis C virus nucleic acid vaccine, WO 2005071093 (A3) chimpanzee adenovirus vaccine carriers, WO 03031588 (A2) hepatitis C virus vaccine]. TH is a named inventor on patent WO06123256. The remaining authors declare that the research was conducted in the absence of any commercial or financial relationships that could be construed as a potential conflict of interest.
