# Supplementary material for: A Novel Vaccine Strategy Employing Serologically Different Chimpanzee Adenoviral Vectors for the Prevention of HIV-1 and HCV Coinfection
Source: Front Immunol. 2019 Jan 18;9:3175. doi: 10.3389/fimmu.2018.03175 (PMC6346592; doi:10.3389/fimmu.2018.03175)
Supplement: Supplementary file 1 [file Data_Sheet_1.docx]

**A**

**
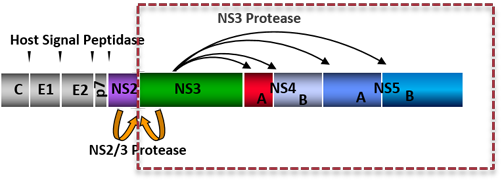
**

**B**

**
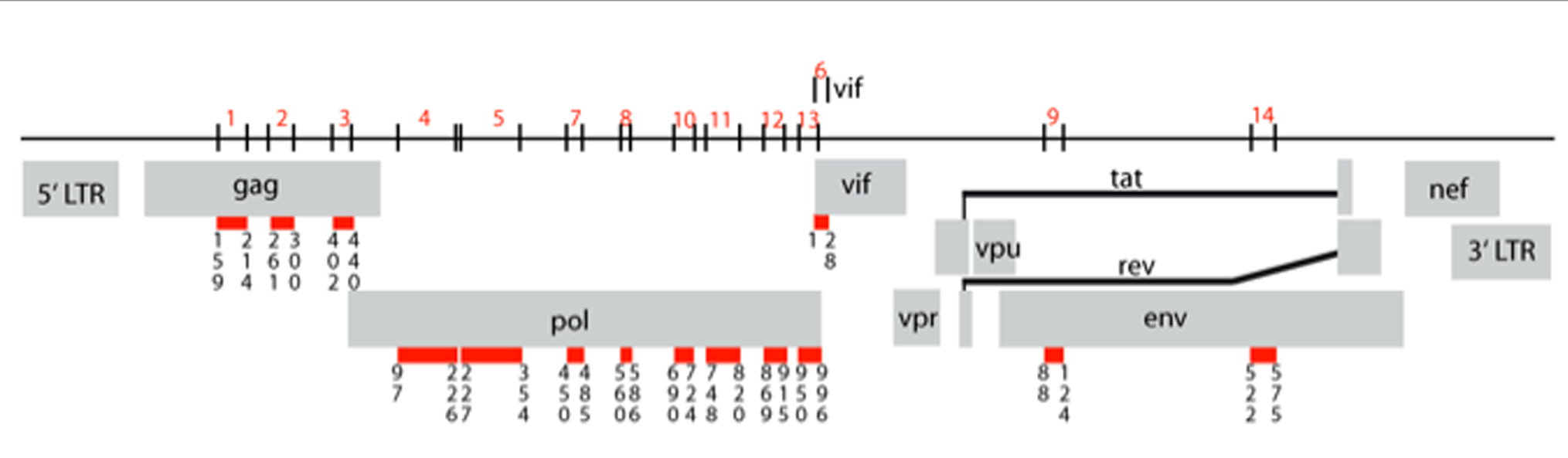
Supplementary Figure 1**

A. The non-structural (NS) region of the hepatitis C virus genome (genotype 1, subtype 1b) encoded by the ChAd3-NSmut and MVA-NSmut vaccines is 1985 amino acids (aa) long and spans NS3, NS4a, NS4b, NS5a, NS5b. Proteolytic cleavage of the HCV polyprotein is mediated by the encoded NS3 protease. This region of NS is highly conserved, with sequence identity of 75-80% among genotypes 1-6. B. The HIVconsv immunogen comprises the 14 most highly conserved regions of the HIV genome (shown in red). Numbers written vertically under each fragment boundary indicate the first and last aa positions using the HXB2 reference strain numbering (adapted from Letourneau S et al. Plos ONE 2007; 2: e984).

**
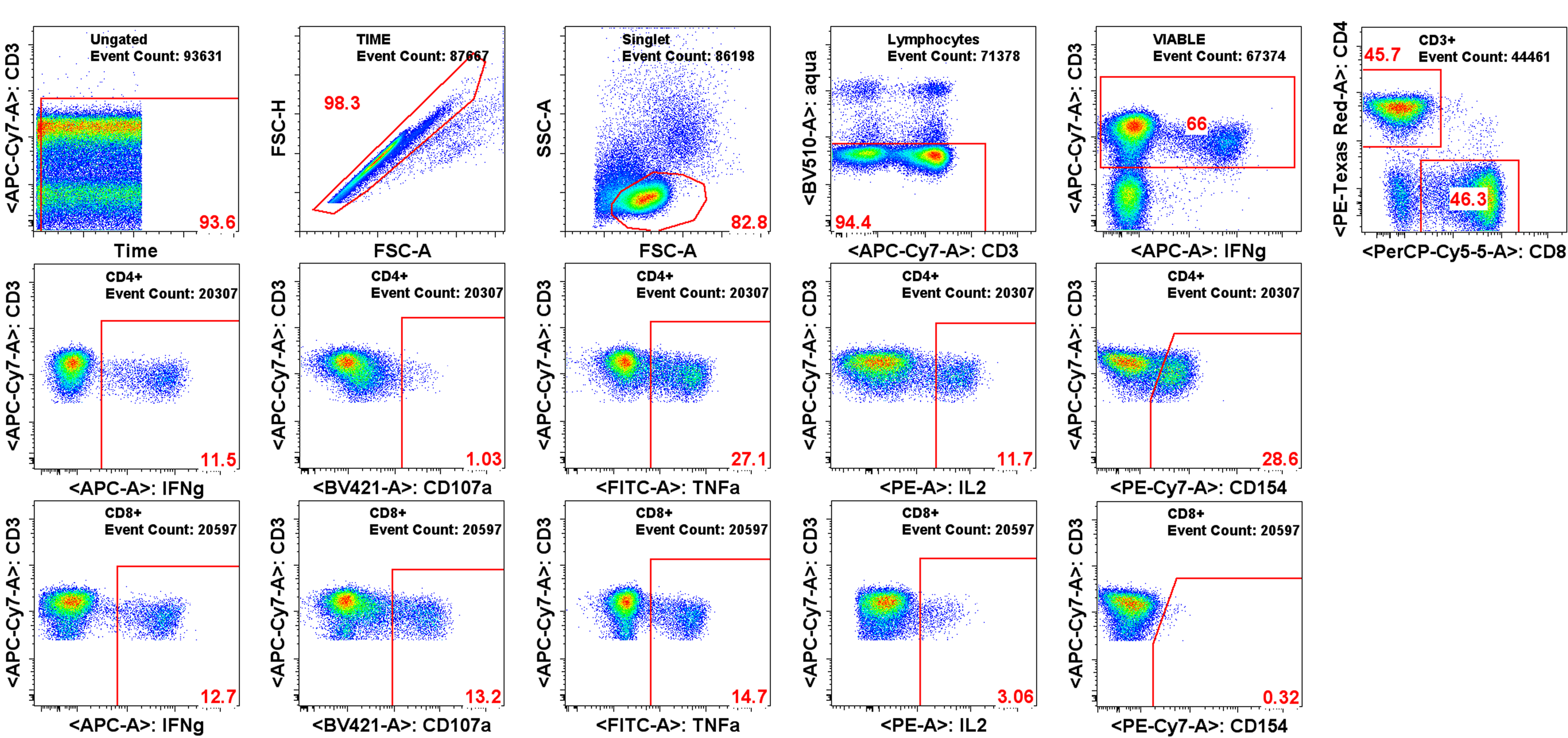
**

**Supplementary Figure 2**

Example staining and gating for identification of antigen-specific CD4+ and CD8+ T cells: viable CD4+ and CD8+ populations were derived from singlet lymphocytes are gated on CD3+ populations; cytokine-, CD40 ligand (CD154)- and CD107a-expressing cells were enumerated by plotting against lineage markers and gating to determine their frequency within the parent population.

**
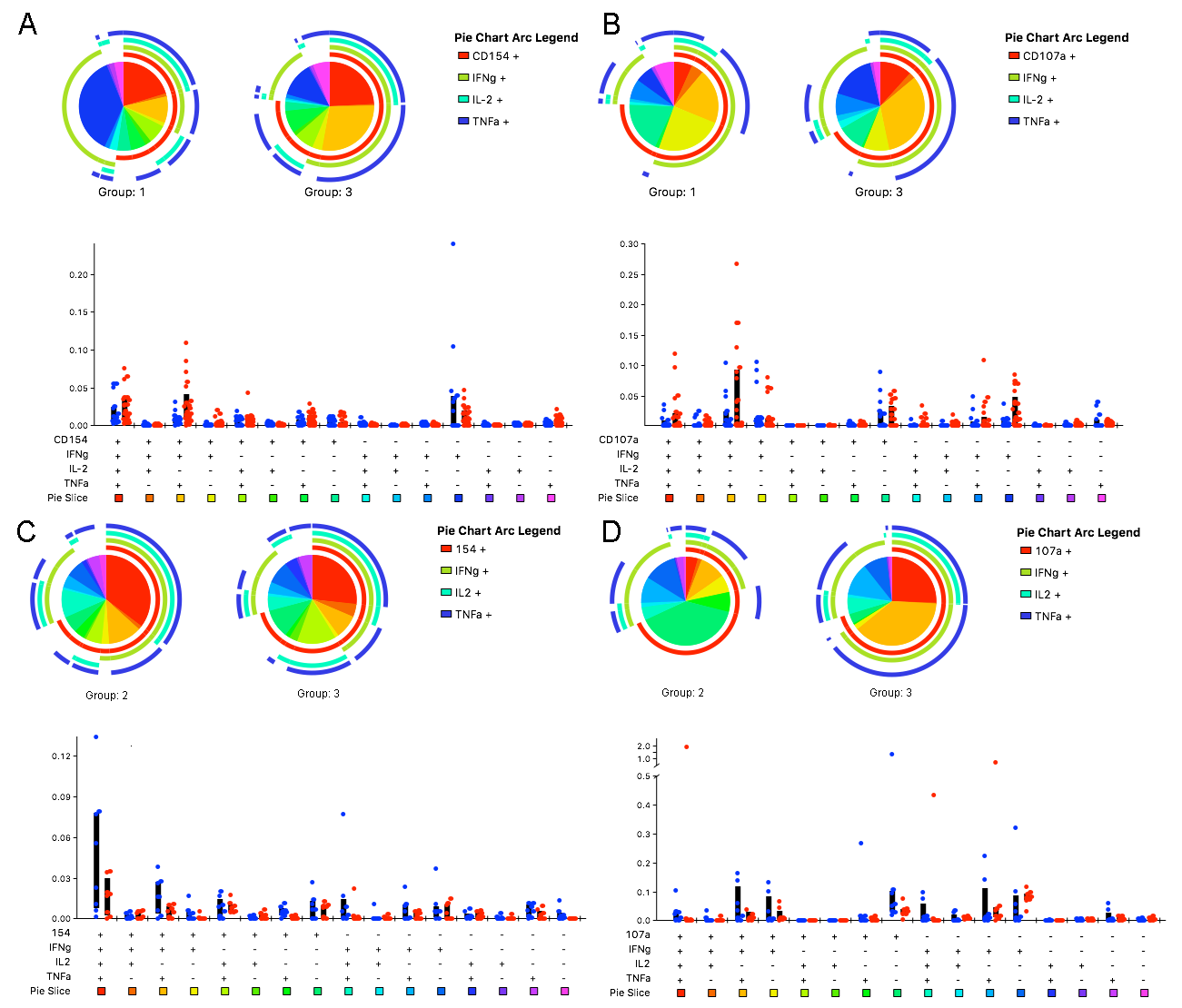
**

**Supplementary Figure 3**

Dot plots show frequencies of antigen-specific CD4+ (A,C) and CD8+ (B,D) T cell populations defined by parameters shown on the x axis. Blue dots represent vaccinees in Group 1 (HCV NSmut – A,B) or Group 2 (HIVconsv – C,D); red dots represent vaccinees in Group 3 (HCV NSmut – top; HIVconsv – bottom). Black bars indicate interquartile ranges. Pie charts show proportions of cells expressing the markers indicated by coloured arcs.

**
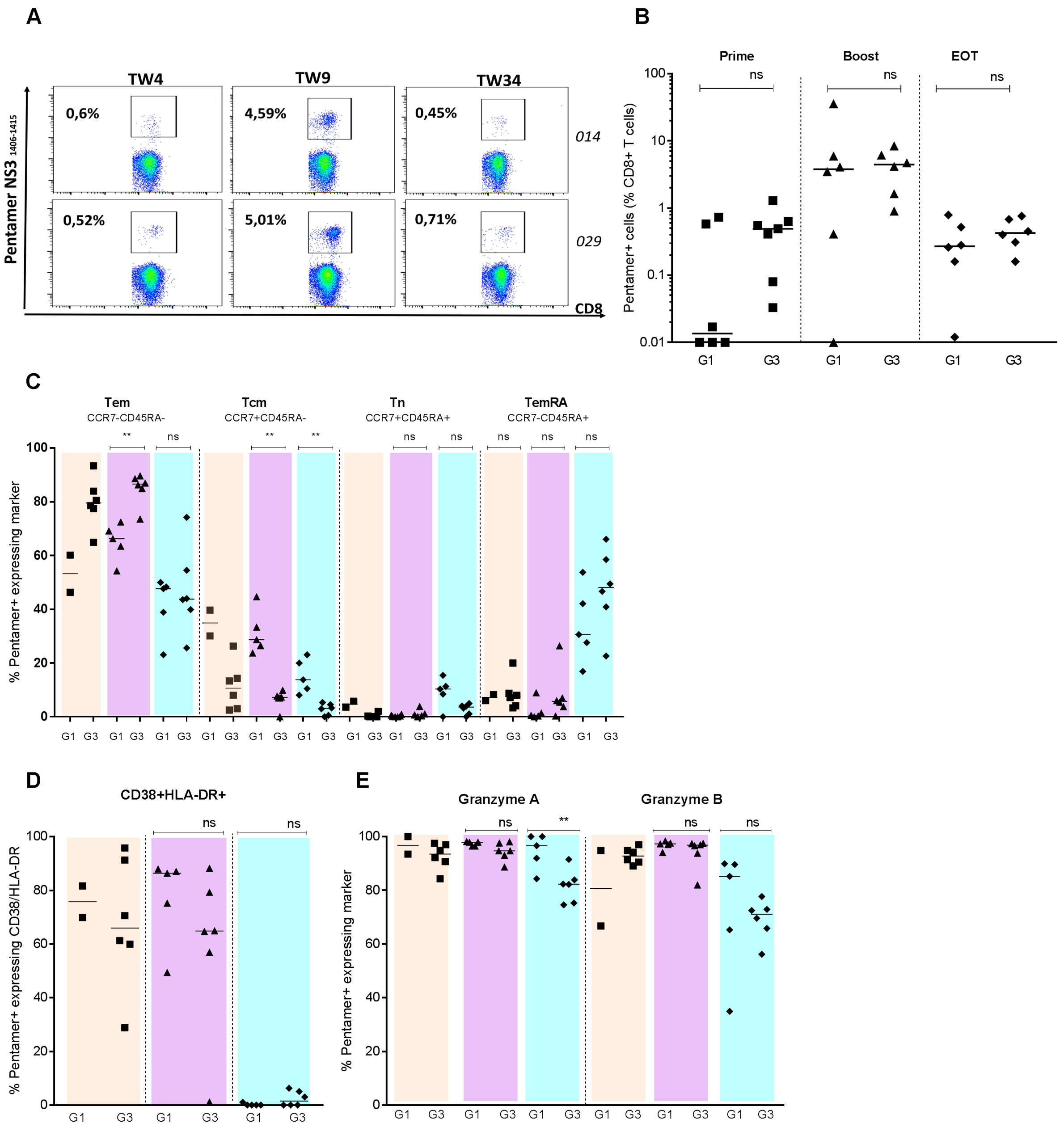
**

**Supplementary Figure 4**

A. Example FACS plot of staining of PBMC ex vivo with pentamer A2/HCV-NS3_1406-1415_ in two representative volunteers 04014 (Group 1) and 04029 (Group 3) after ChAd3-NSmut prime (TW4), MVA-NSmut boost (TW9) and end of trial (TW34). Gating is on live CD3+ cells. The values indicate the percentage of CD8+ T cells binding the pentamer. (B) Frequencies of CD8+ pentamer+ cells (HLA-A2/HCV-NS_1406-1415_ or HLA-A1/HCV-NS_1435-1443_) during the course of the trial are shown for volunteers in Groups 1 and 3 with these HLA class I alleles. (C) Distribution of pentamer+ cells across naïve, Tem, Tcm and TemRA subsets; (D) proportions of pentamer+ cells co-expressing activation markers CD38, HLA-DR and (E) Granzymes A and B (E) at 2-4 weeks after ChAd3-NSmut prime (light pink background), 1-4 weeks after MVA-NSmut boost (light violet background) and at end of trial (light blue background). Horizontal lines indicate medians. P values were determined from Mann-Whitney T test.

 **
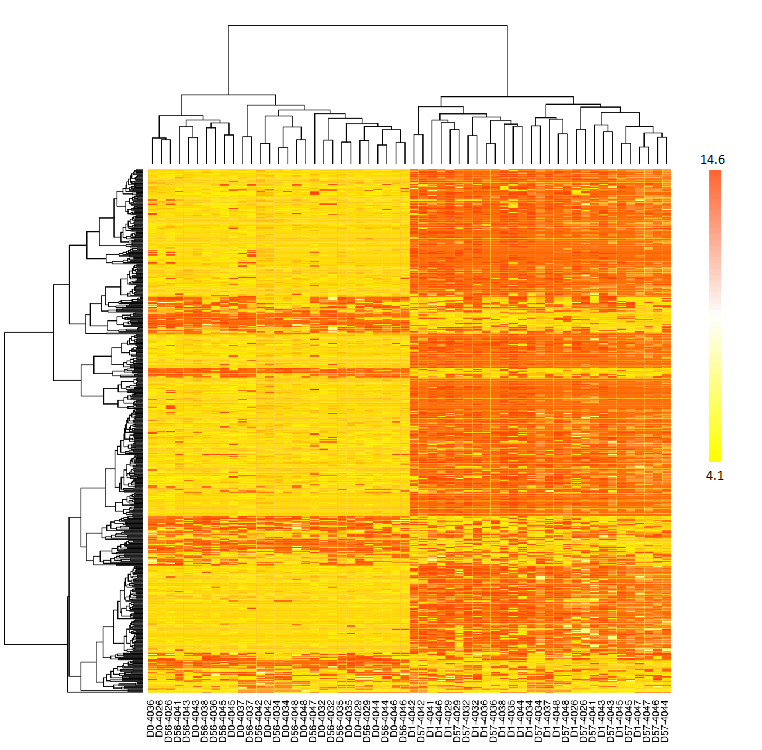
**

**Supplementary Figure 5**

Gene expression profile of Groups 2 (left) and 3 (right) on days 0, 1, 56 and 57. The heatmap depicts differential up- (orange) or down-regulation (yellow) of genes observed 24 hours after vaccination (days 1 and 56) compared to (pre-vaccine administration) days 0 and 56 respectively.

**Supplementary Table 1 Reagent panel for intracellular cytokine analysis**

| **Excitation (nm)** | **Detector** | **Bandpass filter** | **Longpass filter** | **Fluorochrome** | **Marker** | **Clone** | **Dilution** | **Supplier** |
| --- | --- | --- | --- | --- | --- | --- | --- | --- |
| 405 | V525 | 525/50 | 505 | Aqua | live dead | N/A | 1:400 | Invitrogen |
| 640 | R780 | 780/60 | 750 | APC "Fire"750 | CD3 | SK7 | 1:160 | Biolegend |
| 561 | G610 | 610/20 | 595 | Pe "Dazzle"594 | CD4 | RPA T4 | 1:160 | Biolegend |
| 488 | B670 | 670/30 | 655 | PerCP Cy5.5 | CD8 | RPA T8 | 1:160 | Biolegend |
| 405 | V450 | 450/50 | NA | BV421 | CD107a | H4A3 | 1:320 | BD Biosciences |
| 561 | G780 | 780/60 | 750 | PeCy7 | CD154 | 24-31 | 1:320 | Biolegend |
| 640 | R670 | 670/30 | NA | APC | IFNg | B27 | 1:320 | BD Biosciences |
| 561 | G586 | 586/15 | 550 | PE | IL2 | MQ1-17HI2 | 1:20 | BD Biosciences |
| 488 | B530 | 530/30 | 505 | FITC | TNFa | Mab11 | 1:640 | BD Biosciences |

**Supplementary Table 2 Reagent panel for HLA class I-peptide pentamer staining**

| **Excitation (nm)** | **Detector** | **Bandpass filter** | **Longpass filter** | **Fluorochrome** | **Marker** | **Clone** | **Dilution** | | **Supplier** |
| --- | --- | --- | --- | --- | --- | --- | --- | --- | --- |
| 633 | A780 | 780/60 | 755 | NIR | live dead | N/A | 1:1000 | Invitrogen | |
| 405 | D560 | 560/20 | 545 | Pacific orange | CD3 | UCHT1 | 1:50 | Invitrogen | |
| 410 | F450 | 450/50 | NA | Pacific blue | CD8 | RPA T8 | 1:200 | Biolegend | |
| 561 | A780 | 780/60 | 755 | PE-Cy7 | CCR7 | N/A | 1:50 | BD Biosciences | |
| 488 | F530 | 530/30 | 505 | FITC | CD45RA | HI100 | 1:50 | Biolegend | |
| 488 | B695 | 695/40 | 685 | PerCP-Cy5.5 | CD38 | HIT-2 | 1:50 | Biolegend | |
| 633 | B730 | 730/45 | 710 | Alexa Fluor 700 | HLA-DR | G46-6 | 1:50 | BD Biosciences | |
| 488 | B695/40 | 695/40 | 685 | PerCP-Cy5.5 | Granzyme A | CB9 | 1:50 | BD Biosciences | |
| 633 | B730/45 | 730/45 | 710 | Alexa Fluor 700 | Granzyme B | GB11 | 1:50 | BD Biosciences | |
| 561 | E575 | 575/26 | 550 | PE | Pentamer | N/A | 1:50 | Proimmune | |

**Supplementary Table 3 Unsolicited adverse events (clinical) ***

| **Volunteer** | **Group** | **Time point** | **Event** | **Grade** |
| --- | --- | --- | --- | --- |
| 04019 | 2 | Day 57 | Rash on hands | 1 |
| 04020 | 2 | Day 0 | Gastro-intestinal symptoms | 1 |
| 04031 | 2 | Day 28 | Headache | 3 |
| 04031 | 2 | Day 28 | Nasal congestion | 1 |
| 04031 | 2 | Day 57 | Headache | 3 |
| 04031 | 2 | Day 57 | Migraine | 3 |
| 04032 | 3 | Day 28 | Raised blood pressure | 1 |
| 04035 | 3 | Day 7 | Raised blood pressure | 1 |
| 04036 | 3 | Day 56 | Itching hands | 1 |
| 04037 | 3 | Day 57 | Epistaxis | 1 |

* Unsolicited clinical events occurring within 30 days of receipt of ChAd or MVA vaccines. All were considered possibly related to vaccination.

**Supplementary Table 4 Frequency of unsolicited laboratory adverse events***

| **Volunteer** | **Group** | **Time point** | **Parameter** | **Grade** | **Relatedness** |
| --- | --- | --- | --- | --- | --- |
| 04018 | 2 | Day 1 | low lymphocytes | 2 | definitely |
| 04019 | 2 | Day 1 | low lymphocytes | 1 | definitely |
| 04020 | 2 | Day 1 | low lymphocytes | 2 | definitely |
| 04024 | 2 | Day 1 | low lymphocytes | 2 | definitely |
| 04026 | 3 | Day 1 | low lymphocytes | 2 | definitely |
| 04029 | 3 | Day 1 | low lymphocytes | 1 | definitely |
| 04031 | 2 | Day 1 | low lymphocytes | 3 | definitely |
| 04036 | 3 | Day 1 | low lymphocytes | 1 | definitely |
| 04037 | 3 | Day 1 | low lymphocytes | 1 | definitely |
| 04038 | 3 | Day 1 | low lymphocytes | 1 | definitely |
| 04042 | 3 | Day 1 | low lymphocytes | 1 | definitely |
| 04043 | 3 | Day 1 | low lymphocytes | 2 | definitely |
| 04046 | 3 | Day 1 | low lymphocytes | 2 | definitely |
| 04047 | 3 | Day 1 | low lymphocytes | 3 | definitely |
| 04048 | 3 | Day 1 | low lymphocytes | 1 | definitely |
| 04046 | 3 | Day 28 | hypokalaemia | 1 | possibly |
| 04048 | 3 | Day 28 | hypokalaemia | 1 | possibly |
| 04025 | 2 | Day 28 | hyponatraemia | 1 | possibly |
| 04034 | 3 | Day 28 | Raised urea | 1 | possibly |
| 04038 | 3 | Day 28 | low haemoglobin | 2 | possibly |
| 04026 | 3 | Day 57 | low lymphocytes | 1 | definitely |
| 04038 | 3 | Day 57 | low lymphocytes | 2 | definitely |
| 04006 | 1 | Day 84 | hypokalaemia | 1 | possibly |
| 04031 | 2 | Day 28 | hypokalaemia | 1 | possibly |
| 04037 | 3 | Day 84 | hypokalaemia | 1 | possibly |
| 04038 | 3 | Day 84 | hypokalaemia | 1 | possibly |
| 04045 | 3 | Day 84 | low lymphocytes | 1 | definitely |

* Unsolicited laboratory events occurring within 30 days of receipt of ChAd or MVA vaccines.

**Supplementary Table 5**

**Frequencies of NS-specific T cells detected by multiparameter flow cytometry***

|  | G1 prime | G3 prime | G1 boost | G3 boost | G1 EOT | G3 EOT |
| --- | --- | --- | --- | --- | --- | --- |
| **CD8+ cells** | | | | | | |
| IFN-γ |  | | | | | |
| Median (%) | 0.017 | 0.044 | 0.155 | 0.265 | 0.117 | 0.263 |
| IQR | 0.01-0.08 | 0-0.2 | 0.1-0.32 | 0.08-0.75 | 0.02-0.28 | 0.05-0.44 |
| TNF-α |  | | | | | |
| median (%) | 0.026 | 0.001 | 0.095 | 0.204 | 0.07 | 0.2 |
| IQR | 0.01-0.09 | 0-0.04 | 0.03-0.2 | 0.08-0.61 | 0.01-0.15 | 0.01-0.38 |
| IL-2 |  | | | | | |
| median (%) | 0 | 0.002 | 0.007 | 0.049 | 0.006 | 0.062 |
| IQR | 0-0.02 | 0-0.01 | 0-0.09 | 0.03-0.24 | 0-0.09 | 0.01-0.12 |
| CD107 (%) |  | | | | | |
| median | 0.07 | 0.003 | 0.191 | 0.171 | 0.121 | 0.177 |
| IQR | 0.01-0.1 | 0-0.13 | 0.08-0.31 | 0.04-0.62 | 0.09-0.25 | 0.03-0.36 |
| **CD4+ cells** | | | | | | |
| IFN-γ |  | | | | | |
| median (%) | 0.003 | 0.045 | 0.152 | 0.123 | 0.106 | 0.076 |
| IQR | 0-0.02 | 0-0.12 | 0.08-0.24 | 0.07-0.36 | 0.05-0.26 | 0.04-0.1 |
| TNF-α |  | | | | | |
| median (%) | 0.023 | 0.019 | 0.132 | 0.2 | 0.053 | 0.066 |
| IQR | 0.01-0.04 | 0-0.1 | 0.04-0.2 | 0.1-0.35 | 0.01-0.1 | 0.05-0.12 |
| IL-2 |  | | | | | |
| median (%) | 0.019 | 0.01 | 0.107 | 0.08 | 0.044 | 0.035 |
| IQR | 0-0.05 | 0-0.05 | 0.02-0.17 | 0.05-0.18 | 0-0.05 | 0.02-0.07 |
| CD154 |  |  |  |  |  |  |
| median (%) | 0.022 | 0.011 | 0.155 | 0.176 | 0.051 | 0.067 |
| IQR | 0-0.05 | 0-0.11 | 0.03-0.17 | 0.07-0.35 | 0.01-0.09 | 0.03-0.1 |

* Sum of responses to pools F, G, H, I, L, M after subtraction of mock-stimulated values.

**Supplementary Table 6**

**Frequencies of HIVconsv-specific T cells detected by multiparameter flow cytometry***

|  | G2 prime | G3 prime | G2 boost | G3 boost | G2 EOT | G3 EOT |
| --- | --- | --- | --- | --- | --- | --- |
| **CD8+ cells** | | | | | | |
| IFN-γ |  | | | | | |
| median (%) | 0.01 | 0.06 | 0.058 | 0.059 | 0 | 0.07 |
| IQR | 0-0.07 | 0.01-0.15 | 0.01-0.08 | 0-0.18 | 0-0.05 | 0.01-0.27 |
| TNF-α |  | | | | | |
| median (%) | 0.003 | 0.061 | 0.055 | 0.035 | 0 | 0.046 |
| IQR | 0-0.08 | 0-0.08 | 0-0.07 | 0.01-0.09 | 0-0.01 | 0.03-0.12 |
| IL-2 |  | | | | | |
| median (%) | 0.001 | 0.012 | 0.016 | 0.01 | 0 | 0.008 |
| IQR | 0-0.01 | 0-0.02 | 0-0.04 | 0-0.02 | 0-0.02 | 0-0.02 |
| CD107 |  | | | | | |
| median (%) | 0 | 0.029 | 0.065 | 0.016 | 0.025 | 0.013 |
| IQR | 0-0.05 | 0-0.09 | 0.04-0.15 | 0-0.1 | 0-0.06 | 0-0.27 |
| **CD4+ cells** | | | | | | |
| IFN-γ |  | | | | | |
| median (%) | 0.004 | 0.035 | 0.035 | 0.04 | 0.019 | 0.02 |
| IQR | 0-0.05 | 0-0.05 | 0.02-0.11 | 0.01-0.05 | 0-0.02 | 0-0.04 |
| TNF-α |  | | | | | |
| median (%) | 0.004 | 0.02 | 0.05 | 0.045 | 0.017 | 0.025 |
| IQR | 0-0.06 | 0-0.04 | 0.02-0.15 | 0.01-0.05 | 0-0.04 | 0.02-0.03 |
| IL-2 |  | | | | | |
| median (%) | 0.001 | 0.02 | 0.032 | 0.04 | 0.001 | 0.02 |
| IQR | 0-0.03 | 0-0.03 | 0.01-0.09 | 0.01-0.05 | 0-0.01 | 0-0.04 |
| CD154 |  | | | | | |
| median (%) | 0.015 | 0.02 | 0.06 | 0.05 | 0.001 | 0.03 |
| IQR | 0-0.03 | 0-0.03 | 0.03-0.12 | 0.02-0.06 | 0-0.02 | 0.01-0.05 |

* Sum of responses to pools 3 and 4 after subtraction of mock-stimulated values.

**Supplementary Table 7 Significantly enriched Gene Ontology (GO terms) associated with differentially expressed genes (DEGs)**

| **Pathway** | **GO term** | **Number of genes** | **Adjusted p value*** |
| --- | --- | --- | --- |
| **HCV 003 day 1** |  |  |  |
| Response to Virus | GO:0009615 | 40 | 3x10^-34^ |
| IFN I signaling pathway | GO:0060337 | 20 | 4.5x10^-23^ |
| Cytokine-mediated signaling pathway | GO:0019221 | 53 | 7x10^-24^ |
| Negative regulation of viral life cycle | GO:1903901 | 20 | 1x10^-20^ |
| IFN gamma-mediated signaling pathway | GO:0060333 | 15 | 1.7x10^-14^ |
| Response to cytokine | GO:0034097 | 27 | 1.4x10^-11^ |
| Response to IFN alpha | GO:0035455 | 6 | 4x10^-6^ |
| Response to IFN-beta | GO:0035456 | 6 | 8x10^-6^ |
| Response to IFN-gamma | GO:0034341 | 10 | 8.8x10^-6^ |
| Inflammatory response | GO:0006954 | 16 | 5x10^-4^ |
| Cytoplasmic PRR signaling pathway in response to virus | GO:0039528 | 3 | 6x10^-4^ |
| Regulation of IL-10 production | GO:0032653 | 5 | 8x10^-3^ |
| Regulation of viral entry into host cell | GO:0046596 | 4 | 1.4x10^-2^ |
| Regulation of IL-1 beta production | GO:0032651 | 5 | 1.7x10^-2^ |
| Regulation of T cell activation | GO:0050863 | 10 | 3.8x10^-2^ |
| Regulation of I-kappaB kinase/NF-kappaB signaling | GO:0043122 | 8 | 6.4x10^-2^ |
| **HCV 003 day 57** |  |  |  |
| Leukocyte activation | GO:0045321 | 219 | 3.3x10^-43^ |
| Neutrophil activation | GO:0042119 | 157 | 1.8x10^-42^ |
| Cytokine-mediated signaling pathway | GO:0019221 | 190 | 8.4x10^-41^ |
| Response to Virus | GO:0009615 | 99 | 8.5X10^-32^ |
| Regulation of cytokine production | GO:0001817 | 165 | 9.1x10^-32^ |
| IFN-gamma-mediated signaling pathway | GO:0060333 | 44 | 6.0x10^-25^ |
| Ag processing and presentation of peptide Ag via MHC I | GO:0002474 | 48 | 1.1x10^-22^ |
| IFN I signaling pathway | GO:0060337 | 40 | 3.9x10^-22^ |
| Negative regulation of viral life cycle | GO:1903901 | 42 | 2.9x10^-20^ |
| Response to cytokine | GO:0034097 | 118 | 9.1x10^-18^ |
| Inflammatory response | GO:0006954 | 103 | 3.3x10^-15^ |
| Regulation of I-kappaB kinase/NF-kappaB signaling | GO:0043122 | 65 | 1.1x10^-14^ |
| Regulation of T cell activation | GO:0050863 | 75 | 1.9x10^-12^ |
| Response to IFN-gamma | GO:0034341 | 37 | 1.7x10^-10^ |
| TLR receptor signaling pathway | GO:0002224 | 34 | 3.0x10^-10^ |
| Regulation of IL-1 beta production | GO:0032651 | 25 | 1.1x10^-9^ |
| TRIF-dependent TLR signaling pathway | GO:0035666 | 16 | 6.0x10^-8^ |
| Regulation T-cell proliferation | GO:0042129 | 40 | 2.5x10^-8^ |
| Regulation of TNF production | GO:0032680 | 33 | 1.8x10^-7^ |
| Response to IFN alpha | GO:0035455 | 12 | 1.6x10^-6-^ |
| Response to IFN-beta | GO:0035456 | 18 | 2.6x10^-6^ |
| Regulation of B-cell proliferation | GO:0030888 | 20 | 1.4x10^-5^ |
| MyD88-dependent TLR signaling pathway | GO:0002755 | 14 | 1.7x10^-5^ |

**Supplementary Table 7, continued**

| **Pathway** | **GO term** | **Number of genes** | **Adjusted p value** |
| --- | --- | --- | --- |
| **Group 2 day 1** |  |  |  |
| Response to Virus | GO:0009615 | 77 | 8x10^-14^ |
| Neutrophil activation | GO:0042119 | 101 | 8x10^-10^ |
| IFN I signaling pathway | GO:0060337 | 29 | 6x10^-10^ |
| Cytokine-mediated signaling pathway | GO:0019221 | 102 | 1x10^-8^ |
| Response to cytokine | GO:0034097 | 84 | 5x10^-8^ |
| IFN gamma-mediated signaling pathway | GO:0060333 | 29 | 2x10^-7^ |
| Regulation of T cell activation | GO:0050863 | 62 | 1.5x10^-6^ |
| Regulation of I-kappaB kinase/NF-kappaB signaling | GO:0043122 | 62 | 4x10^-6-^ |
| Inflammatory response | GO:0006954 | 79 | 6x10^-5^ |
| Regulation of IL-1 beta production | GO:0032651 | 29 | 4x10^-4^ |
| Response to IFN alpha | GO:0035455 | 10 | 8x10^-4^ |
| Response to IFN-gamma | GO:0034341 | 22 | 2x10^-3^ |
| Regulation of TNF superfamily cytokine production | GO:1903555 | 30 | 2.3x10^-3^ |
| Response to IFN-beta | GO:0035456 | 11 | 7.5x10^-3^ |
| Ag processing and presentation of peptide Ag via MHC I | GO:0002474 | 24 | 9x10^-3^ |
| TLR receptor signaling pathway | GO:0002224 | 20 | 2x10^-2^ |
| **Group 2 day 57** |  |  |  |
| Response to Virus | GO:0009615 | 64 | 7.8x10^-25^ |
| Cytokine-mediated signaling pathway | GO:0019221 | 79 | 5.1x10^-22^ |
| Regulation of cytokine production | GO:0001817 | 84 | 3.6x10^-21^ |
| Response to cytokine | GO:0034097 | 59 | 2.9X10^-17^ |
| IFN I signaling pathway | GO:0060337 | 27 | 2.8x10^-13^ |
| Inflammatory response | GO:0006954 | 60 | 3.8x10^-13^ |
| Leukocyte activation | GO:0045321 | 86 | 2.7x10^-11^ |
| Negative regulation of viral life cycle | GO:1903901 | 27 | 3.4x10^-10^ |
| Neutrophil activation | GO:0042119 | 52 | 2.0x10^-7^ |
| Response to IFN-gamma | GO:0034341 | 19 | 2.4x10^-7^ |
| Ag processing and presentation of peptide Ag via MHC I | GO:0002474 | 22 | 8.6x10^-7^ |
| Regulation of I-kappaB kinase/NF-kappaB signaling | GO:0043122 | 30 | 1.7x10^-6^ |
| Regulation of IFN-beta production | GO:0032648 | 13 | 7.5x10^-6^ |
| Regulation of TNF production | GO:0032680 | 17 | 5.7x10^-5^ |
| Response to IFN-alpha | GO:0035455 | 10 | 1.3x10^-4^ |
| TLR receptor signaling pathway | GO:0002224 | 16 | 3.0x10^-4^ |
| Regulation of T cell activation | GO:0050863 | 30 | 1.1x10^-3^ |
| Response to IFN-beta | GO:0035456 | 9 | 1.3x10^-3^ |
| Regulation of IL-1 beta production | GO:0032651 | 11 | 1.4x10^-3^ |

**Supplementary Table 7, continued**

| **Pathway** | **GO term** | **Number of genes** | **Adjusted p value** |
| --- | --- | --- | --- |
| **Group 3 day 1** |  |  |  |
| Response to Virus | GO:0009615 | 65 | 8.4x10^-23^ |
| Cytokine-mediated signaling pathway | GO:0019221 | 86 | 3.0x10^-19^ |
| IFN I signaling pathway | GO:0060337 | 34 | 2.6x10^-17^ |
| IFN gamma-mediated signaling pathway | GO:0060333 | 33 | 4.0x10^-15^ |
| Negative regulation of viral life cycle | GO:1903901 | 30 | 2.0x10^-13^ |
| Response to cytokine | GO:0034097 | 60 | 5.0x10^-13^ |
| Neutrophil activation | GO:0042119 | 62 | 9.8x10^-11^ |
| Ag processing and presentation of peptide Ag via MHC I | GO:0002474 | 25 | 3.0x10^-7-^ |
| Regulation of I-kappaB kinase/NF-kappaB signaling | GO:0043122 | 37 | 8.8x10^-6^ |
| Inflammatory response | GO:0006954 | 50 | 1.6x10^-5^ |
| Response to IFN-gamma | GO:0034341 | 21 | 2.0x10^-5^ |
| Response to IFN alpha | GO:0035455 | 11 | 4.1x10^-5^ |
| Regulation of T cell activation | GO:0050863 | 40 | 5.4x10^-5^ |
| Regulation of viral entry into host cell | GO:0046596 | 11 | 1.7x10^-4^ |
| Regulation of IL-12 production | GO:0032655 | 12 | 1.7x10^-4^ |
| Regulation of IL-6 production | GO:0032675 | 18 | 2.0x10^-4^ |
| TLR receptor signaling pathway | GO:0002224 | 15 | 2.5x10^-4^ |
| Regulation of TNF superfamily cytokine production | GO:1903555 | 20 | 3.2x10^-4^ |
| Response to IFN-beta | GO:0035456 | 10 | 5.5x10^-4^ |
| Regulation of IL-1 beta production | GO:0032651 | 13 | 1.7x10^-3^ |
| **Group 3 day 57** |  |  |  |
| Regulation of cytokine production | GO:0001817 | 108 | 1.2x10^-17^ |
| Cytokine-mediated signaling pathway | GO:0019221 | 79 | 1.5x10^-16^ |
| Response to Virus | GO:0009615 | 68 | 5.8x10^-16^ |
| Leukocyte activation | GO:0045321 | 119 | 1.8x10^-14^ |
| IFN I signaling pathway | GO:0060337 | 35 | 6.2x10^-13^ |
| Neutrophil activation | GO:0042119 | 79 | 6.8x10^-13^ |
| Negative regulation of viral life cycle | GO:1903901 | 31 | 2.1x10^-10^ |
| Response to cytokine | GO:0034097 | 68 | 3.3x10^-10^ |
| Inflammatory response | GO:0006954 | 69 | 2.2x10^-8^ |
| Regulation of I-kappaB kinase/NF-kappaB signaling | GO:0043122 | 46 | 1.6x10^-7^ |
| Ag processing and presentation of peptide Ag via MHC I | GO:0002474 | 25 | 1.1x10^-5^ |
| Regulation of IL-1 beta production | GO:0032651 | 19 | 1.6x10^-5^ |
| Regulation of TNF production | GO:0032680 | 25 | 3.1x10^-5^ |
| Regulation of T-cell activation | GO:0050863 | 46 | 4.6x10^-5^ |
| Response to IFN-gamma | GO:0034341 | 20 | 2.1x10^-4^ |
| Regulation of IFN-beta production | GO:0032648 | 17 | 5.4x10^-4^ |
| Cytokine production | GO:0001816 | 17 | 2.1x10^-3^ |
| Response to IFN-alpha | GO:0035455 | 10 | 3.1x10^-3^ |
| Response to IFN-beta | GO:0035456 | 9 | 8.0x10^-3^ |

* p values for days 1 and 57 are derived from comparisons with days 0 and 56 respectively
